# Tripartite Dynamic Zero-Sum Quantum Games

**DOI:** 10.3390/e23020154

**Published:** 2021-01-27

**Authors:** Hui-Min Cheng, Ming-Xing Luo

**Affiliations:** Information Security and National Computing Grid Laboratory, Southwest Jiaotong University, Chengdu 610031, China; hm.cheng2021@gmail.com

**Keywords:** quantum game, Nash-equilibrium, fairness, dynamic zero-sum game

## Abstract

The Nash equilibrium plays a crucial role in game theory. Most of results are based on classical resources. Our goal in this paper is to explore multipartite zero-sum game with quantum settings. We find that in two different settings there is no strategy for a tripartite classical game being fair. Interestingly, this is resolved by providing dynamic zero-sum quantum games using single quantum state. Moreover, the gains of some players may be changed dynamically in terms of the committed state. Both quantum games are robust against the preparation noise and measurement errors.

## 1. Introduction

The quantum state as an important resource has been widely used for accomplishing difficult or impossible tasks with classical resources [1]. Quantum game theory as one of important applications is to investigate strategic behavior of agents using quantum resources. It is closely related to quantum computing and Bell theory [2,3,4]. In most cases, distributive tasks can be regarded as equivalent quantum games [5,6]. So far, it has been widely used in Bell tests [3,7], quantum network verification [8], distributed computation [8,9,10,11,12,13], parallel testing [14,15,16,17], device-independent quantum key distribution [18,19,20,21].

Different from those applications, Marinatto and Weber present a two quantum game using Nash strategy which gives more reward than classical [22]. Eisert et al. resolve the prisoner’s dilemma with quantum settings by providing higher gains than its with classical settings [23]. Sekiguchi et al. have prove that the uniqueness of Nash equilibria in quantum Cournot duopoly game [24]. Brassard et al. recast colorredMermin’s multi-player game in terms of quantum pseudo-telepathy [25]. Meyer introduces the quantum strategy for coin flipping game [26]. In the absence of a fair third party, Zhang et al. prove that a space separated two party game can achieve fairness by combining Nash equilibrium theory with quantum game theory [27]. All of these quantum games show different supremacy to classical games. Nevertheless, there are specific games without quantum advantage. One typical example is guessing your neighbor’s input game (GYNI) [28] or its generalization [8]. Hence, it is interesting to find games with different features.

Every game contains three elements: player, strategy and gain function [6,22]. There are various classification according to different benchmarks. According to the participants’ understanding of other participants, one game may be a complete information game [3] or a incomplete information game [28]. From the time sequence of behavior, it may be divided into static game [22] and dynamic game [27,29]. Another case is cooperating game [3] or competing game (non-cooperating game) [22]. Non cooperative game can be further divided into complete information static game, complete information dynamic game, incomplete information static game and incomplete information dynamic game [22,29]. As the basis of non-cooperative game [29], Nash theory is composed of the optimal strategies of all participants such that no one is willing to break the equilibrium. Moreover, it is a zero-sum game if the total gains is zero for any combination of strategies [27]. Otherwise, it is a non zero-sum game. So far, most of games are focused on cooperative games such as Bell game [3]. Our goal in this paper is to find dynamic zero-sum games with Nash equilibrium. Dynamic game refers to that the actions of different players have a sequence, and the post actor can observe the actions chosen by the actor in front of him [29]. Different from bipartite zero-sum game [27], we provide tripartite quantum fair zero-sum games which cannot be realized in classical scenarios even if it is difficult to evaluating Nash equilibrium [30]. The interesting feature is that the present quantum game uses of only clean qubit without entanglement.

The rest of paper is organized as follows. In Section 2, we introduce a tripartite zero-sum game with two different settings inspired by bipartite game [27]. We show that there is no strategy for achieving a fair game using classical resources. Both models can be regarded as complete information dynamic zero-sum game. In Section 3, we present quantum zero-sum games with the same settings using quantum single-photon states. Both games are asymptotically fair in terms of some free parameters. Although any quantum pure state is unitarily equivalent to a classical state, our results show that this kind of resources are also useful for special quantum tasks going beyond classical scenarios. In Section 4, we show that the robustness of two quantum games. The last section concludes the paper.

## 2. Classical Tripartite Dynamic Zero-Sum Games

### 2.1. Game Model

We firstly present some definitions as follows.

**Definition** **1.** *Dynamic zero-sum game means that the actions of different players have a sequence, the later participant can observe the formers actions, and the sum of payments for all players is zero for any combination of strategies.*

**Definition** **2.** *Fair game means that the game does not favor any player. Games in this paper are all zero-sum games, thus the fairness in this paper means everyone’s average gain is zero.*

**Definition** **3.** *Asymptotically fair game means that under the given initial conditions, the game may not be fair, but with the increase of variable parameter, the degree of deviation from the fair game becomes smaller and smaller and the game is fair when the variable parameter approaches infinity.*

**Figure 1:**
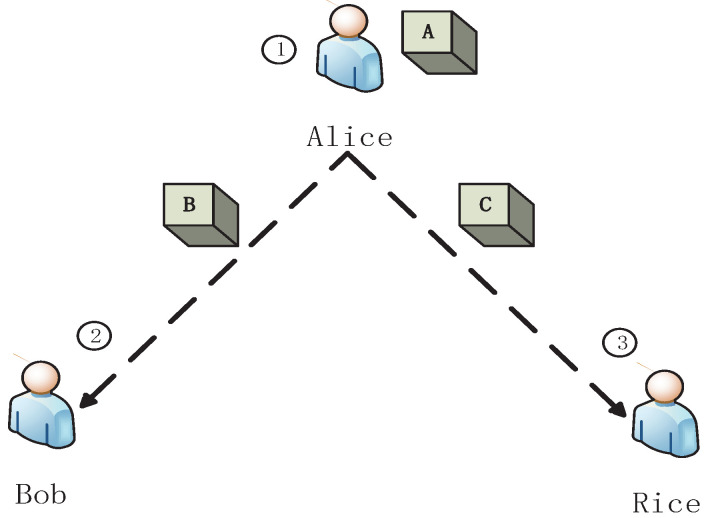
A schematic tripartite dynamic zero-sum classical game G. Alice puts a ball into three boxes ⋄A, ⋄B and ⋄C. And then, she sends ⋄C to Rice, and sends ⋄B to Bob. Rice can choose to open ⋄C or let Alice open ⋄A. If Rice opens ⋄C and there is no ball in it, it is Bob’s turn to open ⋄B or ask Alice to open ⋄A.

Inspired by bipartite game [27], we present a tripartite game G, as shown in Figure 1.

In the present games, we assume that the actions of different participants have a sequence, where the latter participant can observe the former’s action. There are four stages in the present model as follows.

S1. Alice randomly puts a ball into one of three black boxes, ⋄A, ⋄B and ⋄C. Alice sends the box ⋄C to Rice, and sends ⋄B to Bob.
S2. Bob gives his own choice, i.e., he chooses to open ⋄B or asks Alice to open ⋄A, but he does not take any action.
S3. Rice chooses her own strategy and takes action, i.e., she opens ⋄C or lets Alice open ⋄A. If Rice opens ⋄C and there is no ball in the box, the game enters the fourth stage.
S4. It is Bob’s turn to take action according the strategy he has chosen in S2, i.e., he opens box ⋄B or asks Alice to open box ⋄A.

Moreover, for any strategy combination, we assume that the total payment of all participants is zero. In the following, we will explore this kind of games with two settings with different gains.

**Figure 2:**
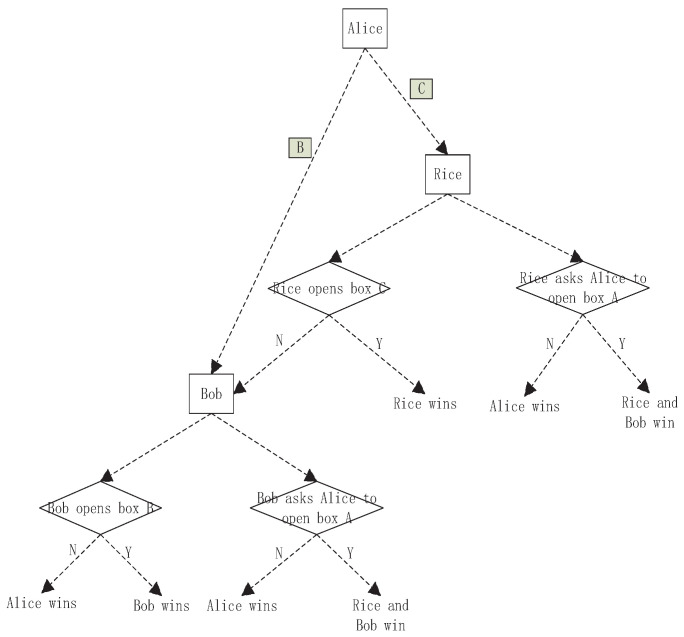
The classical game tree. The rectangle with color represents the black box with the ball while the rectangle without color represents an entity, i.e., Alice, Bob and Rice. The diamond represents an operation. *N* means no ball being found, and *Y* means the ball being found.

The classical game tree is shown in Figure 2. In the stages of G, the wining rules of the game are given by

W1. Bob and Rice win if Rice chooses to open ⋄A and finds the ball in S3.
W2. Alice wins if Rice does not find the ball in box ⋄A in S3.
W3. Rice wins if Rice opens ⋄C and finds the ball in S3.
W4. Bob and Rice win if Bob opens ⋄A and finds the ball in S4.
W5. Alice wins if Bob opens ⋄A and does not find the ball in ⋄A in S4.
W6. Bob wins if Bob opens box ⋄B and finds the ball in S4.
W7. Alice wins if Bob opens box ⋄B and does not find the ball in S4.

Now, for convenience, we define the following probabilities.

P1. Denote $P_{1}$ as the probability that Alice puts the ball into ⋄A.
P2. Denote $P_{2}$ as the probability that Alice puts the ball into ⋄B or ⋄C.
P3. Denote $P_{⋄C}$ as the probability that Rice chooses to open ⋄C.
P4. Denote $P_{⋄B}$ as the probability that Bob chooses to open ⋄B.

Here, we assume that Alice has equal probability to put the ball into ⋄B or ⋄C. It follows that $P_{1}+2P_{2}=1$, with $P_{⋄B},P_{⋄C}\in \left[0,1\right]$.

From winning rules W1–W7 of G, it is easy to get the winning probability $P_{Rice_{1}}$ for Rice from W3 (i.e., Rice finds the ball after opening ⋄C) is given by

(1) $$\begin{array}{c} P_{Rice_{1}}=P_{2}P_{⋄C} \end{array}$$

Moreover, the winning probability for Bob from W6 (i.e., Rice chooses to open ⋄C and does not find the ball, but Bob finds the ball after opening ⋄B) is given by

(2) $$\begin{array}{c} P_{Bob_{1}}=P_{2}P_{⋄C}P_{⋄B}\left(1-P_{2}\right) \end{array}$$

For Alice, there are three subcases W2, W5 and W7 for winning. It follows that

(3) $$\begin{array}{ccc} P_{Alice} & = & 1-P_{1}+P_{⋄B}P_{⋄C}P_{2}^{2}-P_{⋄B}P_{⋄C}P_{2}-P_{⋄C}P_{2}\\ & & +P_{⋄B}P_{⋄C}P_{1}+P_{⋄C}P_{1}P_{2}-P_{⋄B}P_{⋄C}P_{1}P_{2} \end{array}$$

The winning probability for Bob from W1 and W4 is given by

(4) $$\begin{array}{ccc} P_{Bob_{2}} & = & P_{1}-P_{⋄B}P_{⋄C}P_{1}-P_{⋄C}P_{1}P_{2}\\ & & +P_{⋄B}P_{⋄C}P_{1}P_{2} \end{array}$$

The same result holds for Rice from W1 and W4, i.e., $P_{Rice_{2}}=P_{Bob_{2}}$.

Here, we analyze players’ strategies for the present game G to show the main idea. For Alice, she does not know which box Rice or Bob would choose to open before she prepares. Alice may lose the game if she puts the ball in one of the three boxes with a higher probability. Thus, there is a tradeoff for Alice to choose her strategy (the probability $P_{1}$). For Bob, he does not know which box Rice would to open when he gives the probability $P_{⋄B}$. So, he should consider a known parameter $P_{1}$ given by Alice and an unknown parameter $P_{⋄C}$ given by Rice. Similar analysis can be applied to Rice’s strategy, Rice needs to choose her own parameter based on what she knows before she takes action.

### 2.2. The First Tripartite Classical Game

It is well known that every player will maximize its own interests in a non-cooperative game. In this section, we present the first game $G_{1}$ with the gain setting given in Table 1. Our goal is to show the no-fairness of this game with classical resources.

#### 2.2.1. The Average Gain of Rice

From Table 1, the average gain of Rice is given by

(5) $$\begin{array}{ccc} G_{Rice} & = & 2P_{Rice_{1}}-P_{Bob_{1}}-P_{Alice}+\eta P_{Rice_{2}}\\ & = & 3P_{2}P_{⋄C}+\left(\eta +1\right)P_{1}-\left(\eta +1\right)P_{1}P_{⋄B}P_{⋄C}\\ & & -\left(\eta +1\right)P_{1}P_{2}P_{⋄C}+\left(\eta +1\right)P_{1}P_{2}P_{⋄B}P_{⋄C}-1 \end{array}$$

From Equation (6), we get that the partial derivative of $G_{Rice}$ with respect to $P_{⋄C}$ is given by

(6) $$\begin{array}{ccc} \frac{\partial G_{Rice}}{\partial P_{⋄C}} & = & 3P_{2}-\left(\eta +1\right)P_{1}P_{⋄B}\\ & & +\left(\eta +1\right)P_{⋄B}P_{1}P_{2}-\left(\eta +1\right)P_{1}P_{2} \end{array}$$

From Equation (6), if $\frac{\partial G_{Rice}}{\partial P_{⋄C}}<0$, $G_{Rice}$ is a deceasing function. In this case, Rice has to set $P_{⋄C}=0$ to maximize her gains. If $\frac{\partial G_{Rice}}{\partial P_{⋄C}}>0$, i.e., $G_{Rice}$ is increasing function, Rice will choose $P_{⋄C}=1$ to maximize her gains. Moreover, when $\frac{\partial G_{Rice}}{\partial P_{⋄C}}=0$, i.e., $G_{Rice}$ is a constant, $P_{⋄C}$ can be any probability.

#### 2.2.2. The Average Gain of Bob

Similar to Equation (6), we get that the average gain of Bob is given by

(7) $$\begin{array}{ccc} G_{Bob} & = & -P_{Rice_{1}}+2P_{Bob_{1}}-P_{Alice}+P_{Bob_{2}}\\ & = & 3P_{⋄B}P_{⋄C}P_{2}-3P_{⋄B}P_{⋄C}P_{2}^{2}+2P_{1}\\ & & -2P_{⋄B}P_{⋄C}P_{1}-2P_{⋄C}P_{1}P_{2}\\ & & +2P_{⋄B}P_{⋄C}P_{1}P_{2}-1 \end{array}$$

There are several cases to maximize $G_{Bob}$. We only present one case in the following for explaining the main idea. The other cases are included in Appendix A.

For $\frac{\partial G_{Rice}}{\partial P_{⋄C}}\leq 0$, i.e., $\frac{3P_{2}-\left(\eta +1\right)P_{1}P_{2}}{\left(\eta +1\right)P_{1}\left(1-P_{2}\right)}\leq P_{⋄B}\leq 1$, we get that $P_{⋄C}=0$. From Equation (8), we obtain

(8) $$\begin{array}{c} G_{Bob_{1}}=2P_{1}-1 \end{array}$$

C1. If $0\leq \frac{3P_{2}-\left(\eta +1\right)P_{1}P_{2}}{\left(\eta +1\right)P_{1}\left(1-P_{2}\right)}\leq 1$, we get that $\frac{3}{2\eta +5}\leq P_{1}\leq \frac{3}{\eta +1}$. In this case, Bob chooses $G_{Bob_{1}}$ such that $P_{⋄B}=X_{0}$, where $X_{0}>\frac{3P_{2}-\left(\eta +1\right)P_{1}P_{2}}{\left(\eta +1\right)P_{1}\left(1-P_{2}\right)}$.
C2. If $\frac{3P_{2}-\left(\eta +1\right)P_{1}P_{2}}{\left(\eta +1\right)P_{1}\left(1-P_{2}\right)}<0$, i.e., $\frac{3}{\eta +1}<P_{1}\leq 1$. Owing to $P_{⋄B}\geq 0$, we get that $P_{⋄B}$ can be any probability.

All the results are given in Table 2.

#### 2.2.3. The Average Gain of Alice

In this subsection, we calculate the average gain of Alice, which is denoted by $G_{Alice}$. It is easy to write the expression for $G_{Alice}$ according to Table 1 as follows.

(9) $$\begin{array}{ccc} G_{Alice} & = & -P_{Rice_{1}}-P_{Bob_{1}}+2P_{Alice}-\left(\eta +1\right)P_{Rice_{2}}\\ & = & -3P_{⋄C}P_{2}-3P_{⋄B}P_{⋄C}P_{2}+3P_{⋄B}P_{⋄C}P_{2}^{2}\\ & & +\left(\eta +3\right)P_{⋄C}P_{1}P_{2}+\left(\eta +3\right)P_{⋄B}P_{⋄C}P_{1}\\ & & -\left(\eta +3\right)P_{1}-\left(\eta +3\right)P_{⋄B}P_{⋄C}P_{1}P_{2}+2 \end{array}$$

For the case of $0\leq P_{1}<\frac{3}{2\eta +5}$, it follows from Table 2 that $P_{⋄C}=1$ and $P_{⋄B}=1$. Equation (9) is then rewritten into

(10) $$\begin{array}{c} G_{Alice}=3P_{2}^{2}-6P_{2}+2 \end{array}$$

where $P_{2}=\frac{1-P_{1}}{2}$. It is easy to prove that $G_{Alice}$ achieves the maximum when $P_{1}=\frac{3}{2\eta +5}$. Denote $P_{1}=\frac{3}{2\eta +5}-\epsilon$, where ϵ is a small constant satisfying ϵ>0. It follows from Equation (10) that

(11) $$\begin{array}{c} G_{Alice}=\frac{-\eta^{2}+4\eta +23}{{\left(2\eta +5\right)}^{2}}+O\left(\epsilon \right) \end{array}$$

By using the same method for the rest of cases (see Appendix B for details), we can get Table 3. From Table 3, we get that $\frac{-\eta^{2}+4\eta +23}{{\left(2\eta +5\right)}^{2}}+O\left(\epsilon \right)>\frac{\eta +1}{2\eta +5}$ for 1≤η<2, and $\frac{-\eta^{2}+4\eta +23}{{\left(2\eta +5\right)}^{2}}+O\left(\epsilon \right)<\frac{\eta +1}{2\eta +5}$ for η≥2. Thus, Alice will choose $P_{1}=\frac{3}{2\eta +5}-\epsilon$ to maximize her gain when 1≤η<2, while $P_{1}=\frac{3}{2\eta +5}$ when η≥2.

#### 2.2.4. Fair Zero-Sum Game

From Section 2.2.3, we get that $P_{1}=\frac{3}{2\eta +5}-\epsilon$ for 1≤η<2. By using induction we know that $P_{⋄B}=1$ and $P_{⋄C}=1$ from Table 2. From Equations (6), (8) and (9), the expressions of $G_{Alice}$, $G_{Bob}$ and $G_{Rice}$ with respect to η are shown in Table 4.

Result 1 The tripartite game $G_{1}$ is unfair for any η≥1.

**Figure 3:**
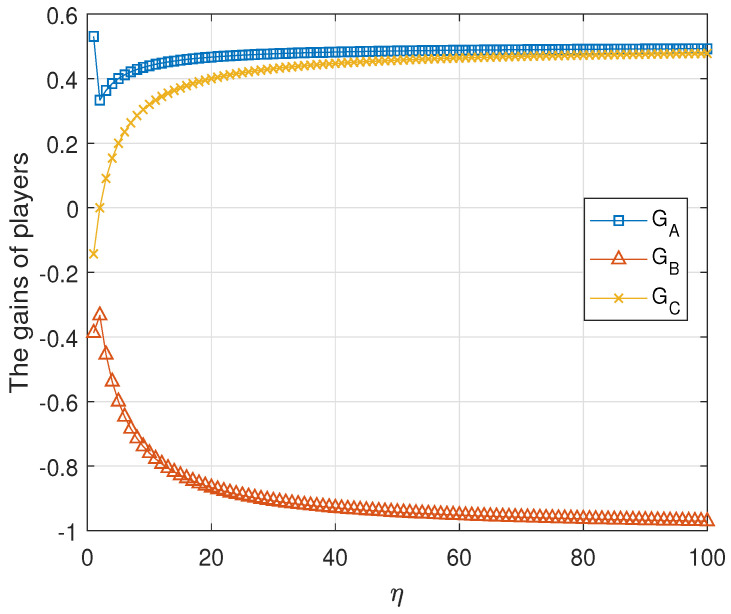
The average gains of three parties in the first classical game $G_{1}$. In this simulation, we assume η≤100 and $\epsilon =10^{-5}$. The gain of Alice is strictly larger than the gains of Bob and Rice for any η≥1.

**Proof.**  The numeric evaluations of $G_{Alice}$, $G_{Bob}$ and $G_{Rice}$ are shown in Figure 3 for η≤100 and $\epsilon =10^{-5}$. It shows that the tripartite classical game $G_{1}$ is unfair. Formally, since the tripartite game $G_{1}$ is a zero-sum game, i.e., the summation of the average gains of all players is zero. It is sufficient to prove that the gain of one player is strictly greater than that of the other. The proof is completed by two cases.

C1. For 1≤η<2, from Table 4, we get that
  (12) $$\begin{array}{c} G_{Alice}-G_{Bob}=\frac{9\eta +36}{{\left(2\eta +5\right)}^{2}}+O\left(\epsilon \right)>0 \end{array}$$
C2. For η≥2, from Table 4, we obtain that
  (13) $$\begin{array}{c} G_{Alice}-G_{Bob}=\frac{3\eta }{2\eta +5}>0 \end{array}$$

if ϵ is very small. This completes the proof. □

### 2.3. The Second Tripartite Classical Game

In this section, we present the second game $G_{2}$ with different settings shown in Table 5. The first game and the second game are the same except for the gain table, i.e., both games adopt the game model in Section 2.1. Similar to the first game $G_{1}$, our goal is to prove its unfair.

Similar to Section 2.2, we can evaluate the gains of all parties, as shown in Table 6. The details are shown in Appendix C, Appendix D and Appendix E. From Table 6, we can prove the following theorem.

Result 2 The tripartite classical game $G_{2}$ is unfair for any μ≥2.

**Figure 4:**
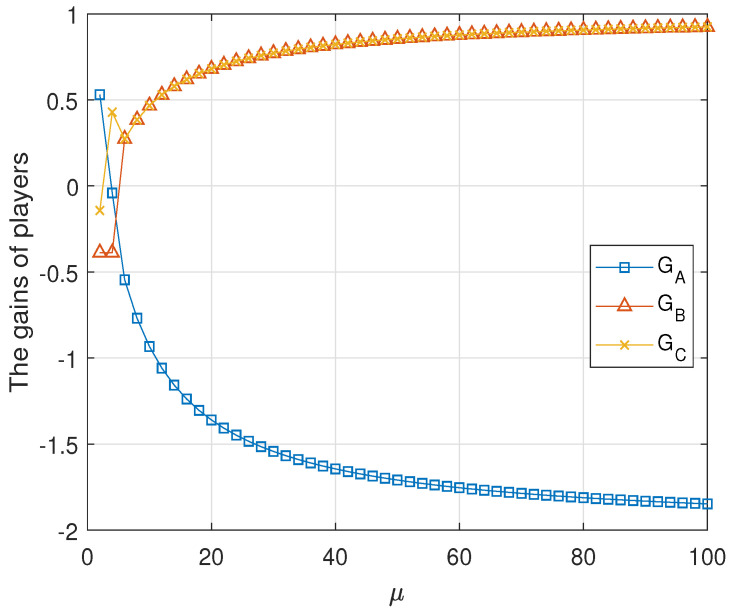
The average gains of three parties in the second classical game $G_{2}$. Here, μ≤100. $G_{Alice}$ and $G_{Bob}$ and $G_{Rice}$ have no common intersection. Moreover, when $\mu >\mu_{1}$, $G_{Bob}$ and $G_{Rice}$ coincide, but do not intersect with $G_{Alice}$.

**Proof.**  The numeric evaluations of $G_{Alice}$, $G_{Bob}$ and $G_{Rice}$ are shown in Figure 4 for μ≤100. It shows that the tripartite classical game $G_{2}$ is unfair. This can be proved formally as follows. From the assumption, the second classical game $G_{2}$ is zero-sum. It is sufficient to prove that there is no μ such that $G_{Alice}$, $G_{Bob}$ and $G_{Rice}$ equal to zero. The proof is completed by two cases.

C1. For $2\leq \mu \leq \frac{41+\sqrt{1345}}{14}$, from Table 6, we get that
  (14) $$\begin{array}{c} G_{Bob}=-\frac{19}{49}\neq 0 \end{array}$$
C2. For $\mu >\frac{41+\sqrt{1345}}{14}$, from Table 6, we obtain that
  (15) $$\begin{array}{c} G_{Bob}=\frac{\mu -3}{\mu +5}\neq 0 \end{array}$$

This completes the proof. □

## 3. Zero-Sum Quantum Games

**Figure 5:**
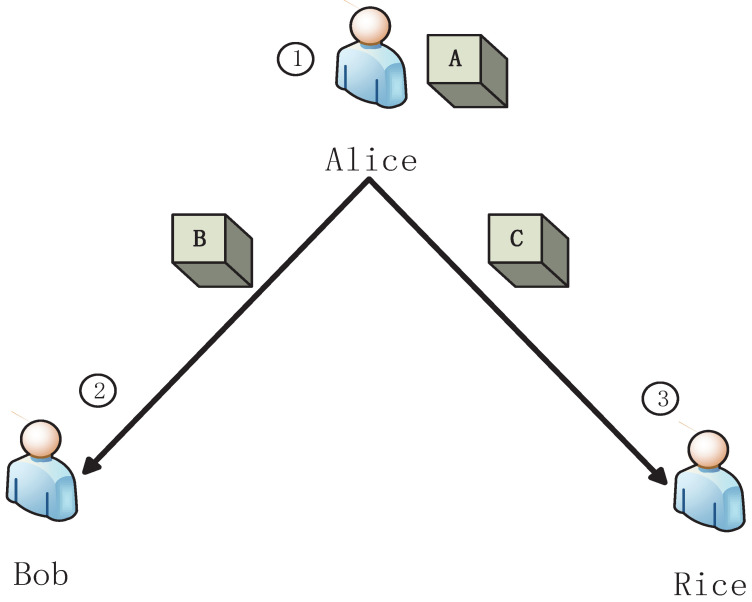
The schematic model of tripartite dynamic zero-sum quantum game. Here, three parties use a single photon to complete the game while the classic game uses a ball in the game given in Figure 1.

In this section, by quantizing the classical game shown in Figure 1, we get that there are also four stages in quantum game, as shown in Figure 5. The correspondence between the classical and quantum game are: the classical game is to put a ball into three ordinary black boxes, while the quantum game is to put a particle into three quantum boxes. In the classical games, Bob and Rice can selectively let Alice open the box ⋄A to prevent Alice from putting the ball into the box ⋄A so that Bob and Rice cannot find the ball. In quantum game, they can prevent this same problem by setting the committed state before the game, i.e., Alice, Bob and Rice agree on which state Alice should set the photon to. Alice has three quantum boxes, ⋄A, ⋄B and ⋄C used to store a photon. The state of the photon in the boxes are denoted |a〉, |b〉 and |c〉. The quantum game is given by the following four stages S1–S4.

S1. Alice randomly puts the single photon into one of the three quantum boxes, ⋄A, ⋄B and ⋄C. Alice sends the box ⋄C to Rice, and sends ⋄B to Bob.
S2. Bob gives his own strategy, and he opens box ⋄B.
S3. Rice chooses her own strategy and takes action, i.e., she opens ⋄C. If neither Rice nor Bob finds the photon, the game enters the fourth stage.
S4. Bob(Rice) asks Alice to send him(her) box ⋄A to verify whether the state of photon prepared by Alice is the same as the committed state.

Moreover, for any strategy combination, we assume that the total payment of all participants is zero and the latter participant can observe the former’s action. In the following, we will explore this kind of games with two settings with different gains.

**Figure 6:**
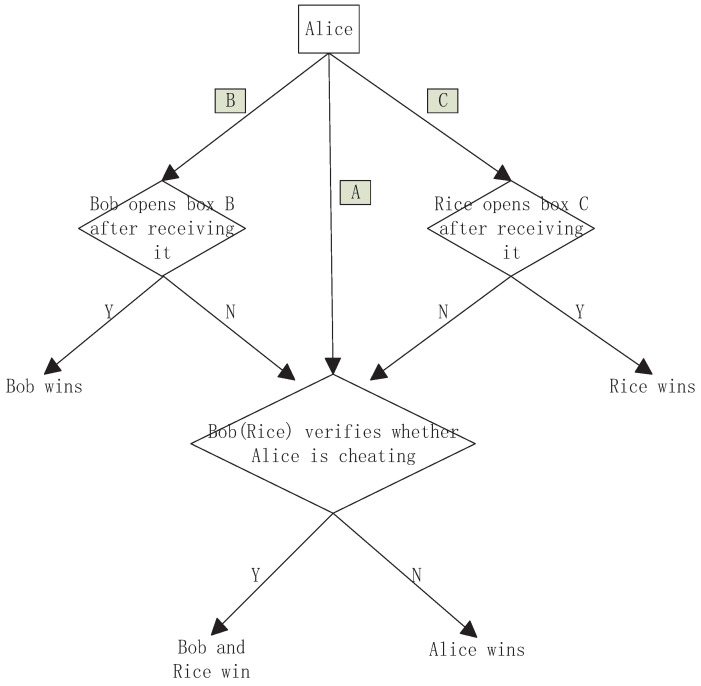
The quantum game tree. The rectangle with color represents the black box with the photon while the rectangle without color represents an entity Alice. The diamond represents an operation. *N* means no photon being found, and *Y* means the photon being found.

The quantum game tree is shown in Figure 6. In the stages of G, the wining rules of the game is given by

W1. Rice wins if Rice finds the photon after opening ⋄C.
W2. Bob wins if Bob finds the photon after opening ⋄B.
W3. Bob and Rice win if neither Rice nor Bob finds the photon but Alice is not honest.
W4. Alice wins if neither Rice nor Bob finds the photon and Alice is honest.

We consider the dynamic zero-sum quantum game with the same setting parameters given in Table 1 and Table 5, but the difference is that each symbol has a slightly different meaning, i.e., in quantum games, $R_{succ}\left(⋄C\right)$ means that Rice finds the photon after opening the box ⋄C. $B_{succ}\left(⋄B\right)$ means that Bob finds the photon after opening the box ⋄B. ${R/B}_{fail}\left(\cdot \right)$ means that neither Rice nor Bob finds the photon and Alice is honest. ${R/B}_{succ}\left(⋄A\right)$ means that neither Rice nor Bob finds the photon but Alice is not honest.

### 3.1. The Winning Rules of The Quantum Game

The winning rules of quantum game is similar to W1–W4. For convenience we define the following probabilities.

P1. Denote $\alpha_{1}$ as the probability that Alice puts the photon into the box ⋄A.
P2. Denote $\alpha_{2}$ as the probability that Alice puts the photon into box ⋄B or box ⋄C.
P3. Denote 1−γ as the probability that Rice chooses to open the box ⋄C when she received ⋄C.
P4. Denote 1−β as the probability that Bob chooses to open the box ⋄B when he received ⋄B.

Similar to classical game shown in Figure 1, we have $\alpha_{1}+2\alpha_{2}=1$ and γ,β∈[0,1].

In quantum scenarios, the box of ⋄A, ⋄B, or ⋄C is realized by a quantum state |a〉,|b〉 or |c〉. The statement of one party finding the photon by opening one box (⋄A for example) means that one party find the photon in the state |a〉 after projection measurement under the basis {|a〉,|b〉,|c〉}. With these assumption, we get an experimental quantum game as follows.

Alice’s preparation. Alice prepares the single photon in the following supposition state

(16) $$\begin{array}{c} |\psi \rangle =\sqrt{\alpha_{1}}|a\rangle +\sqrt{\alpha_{2}}|b\rangle +\sqrt{\alpha_{2}}|c\rangle \end{array}$$

where |a〉,|b〉 and |c〉 can be realized by using different paths, $\alpha_{1}+2\alpha_{2}=1$ and $\alpha_{1}\in \left[0,1\right]$ is a parameter controlled by Alice.

Bob’s operation. Bob splits the box ⋄B into box ⋄B and box $⋄B^{\prime }$ according the following transformation

(17) $$\begin{array}{c} |b\rangle \mapsto \sqrt{1-\beta }|b\rangle +\sqrt{\beta }|b^{\prime }\rangle \end{array}$$

where β∈[0,1] is a parameter controlled by Bob.

Rice’s operation. Rice splits the box ⋄C into two parts ⋄C and $⋄C^{\prime }$ according to the following transformation

(18) $$\begin{array}{c} |c\rangle \mapsto \sqrt{1-\gamma }|c\rangle +\sqrt{\gamma }|c^{\prime }\rangle \end{array}$$

where γ∈[0,1] is a parameter controlled by Rice.

Similar to classical games, Alice may choose a large $\alpha_{1}$ to increase the probability of the photon appearing in box ⋄A, which will then reduce the probability of Bob and Rice finding the photon. However, this strategy may result in losing the game with a high probability for Alice in the verification stage. Similar intuitive analysis holds for others. Hence, it should be important to find reasonable parameters for them. We give the detailed process in the following.

Suppose that Alice prepares the photon in the following state

(19) $$\begin{array}{c} |\psi \rangle =\sqrt{\alpha_{1}}|a\rangle +\sqrt{\alpha_{2}}|b\rangle +\sqrt{\alpha_{2}}|c\rangle \end{array}$$

using path encoding. When Bob (Rice) receives its box ⋄B(⋄C) and splits it into two parts, the final state of the photon is given by

(20) $$\begin{array}{ccc} |\psi_{0}\rangle & = & \sqrt{\alpha_{1}}|a\rangle +\sqrt{\alpha_{2}\left(1-\beta \right)}|b\rangle +\sqrt{\alpha_{2}\beta }|b^{\prime }\rangle \\ & & +\sqrt{\alpha_{2}\left(1-\gamma \right)}|c\rangle +\sqrt{\alpha_{2}\gamma }|c^{\prime }\rangle \end{array}$$

From Equation (20), the probability that Rice finds the photon in ⋄C (using single photon detector) is

(21) $$\begin{array}{c} P_{Rice_{1}}={\left|\langle c|\psi_{0}\rangle \right|}^{2}=\alpha_{2}\left(1-\gamma \right) \end{array}$$

Moreover, the probability that Bob finds the photon in ⋄B is given by

(22) $$\begin{array}{c} P_{Bob_{1}}={\left|\langle b|\psi_{0}\rangle \right|}^{2}=\alpha_{2}\left(1-\beta \right) \end{array}$$

If neither Rice nor Bob finds the photon, the state in Equation (20) will collapse into

(23) $$\begin{array}{ccc} |\psi_{1}\rangle & = & \sqrt{\frac{\alpha_{1}}{\alpha_{1}+\alpha_{2}\beta +\alpha_{2}\gamma }}|a\rangle +\sqrt{\frac{\alpha_{2}\beta }{\alpha_{1}+\alpha_{2}\beta +\alpha_{2}\gamma }}|b^{\prime }\rangle \\ & & +\sqrt{\frac{\alpha_{2}\gamma }{\alpha_{1}+\alpha_{2}\beta +\alpha_{2}\gamma }}|c^{\prime }\rangle \end{array}$$

If Alice did prepare the photon in the committed state $|\phi \rangle =\sqrt{\omega_{1}}|a\rangle +\sqrt{\omega_{2}}|b\rangle +\sqrt{\omega_{2}}|c\rangle$ initially, it is easy to prove that the state at this stage should be

(24) $$\begin{array}{ccc} |\psi_{C}\rangle & = & \sqrt{\frac{\omega_{1}}{\omega_{1}+\omega_{2}\beta +\omega_{2}\gamma }}|a\rangle +\sqrt{\frac{\omega_{2}\beta }{\omega_{1}+\omega_{2}\beta +\omega_{2}\gamma }}|b^{\prime }\rangle \\ & & +\sqrt{\frac{\omega_{2}\gamma }{\omega_{1}+\omega_{2}\beta +\omega_{2}\gamma }}|c^{\prime }\rangle \end{array}$$

where $\omega_{1}+2\omega_{2}=1$. By performing a projection measurement on $|\psi_{1}\rangle$, Rice or Bob gets an ideal state $|\psi_{C}\rangle$ with the probability

(25) $$\begin{array}{c} |\langle \psi_{1}|\psi_{C}\rangle {|}^{2}=\frac{{\left(\sqrt{\alpha_{1}\omega_{1}}+x\sqrt{\alpha_{2}\omega_{2}}\right)}^{2}}{\left(\alpha_{1}+\alpha_{2}x\right)\left(\omega_{1}+\omega_{2}x\right)} \end{array}$$

where x=β+γ.

The probability that neither Rice nor Bob finds the photon when Alice did prepare the photon in the committed state |ϕ〉 is give by

(26) $$\begin{array}{ccc} P_{Alice} & = & \left(1-P_{Rice_{1}}-P_{Bob_{1}}\right){\left|\langle \psi_{1}|\psi_{C}\rangle \right|}^{2}\\ & = & \frac{{\left(\sqrt{\alpha_{1}\omega_{1}}+x\sqrt{\alpha_{2}\omega_{2}}\right)}^{2}}{\omega_{1}+\omega_{2}x} \end{array}$$

Moreover, the probability that neither Rice nor Bob finds the photon but Rice or Bob detects forge state prepared by Alice is denote by $P_{Rice_{2}}$ or $P_{Bob_{2}}$, which is given by

(27) $$\begin{array}{ccc} P_{Rice_{2}} & = & \left(1-P_{Rice_{1}}-P_{Bob_{1}}\right)\left(1-\right|\langle \psi_{1}|\psi_{C}\rangle {|}^{2})\\ & = & -\frac{{\left(\sqrt{\alpha_{1}\omega_{1}}+x\sqrt{\alpha_{2}\omega_{2}}\right)}^{2}}{\omega_{1}+\omega_{2}x}+\alpha_{1}+\alpha_{2}x \end{array}$$

with $P_{Rice_{2}}=P_{Bob_{2}}$ from winning rule W3.

### 3.2. The First Tripartite Quantum Game

In this subsection, we introduce the quantum implementation of the first game $G_{1}$ with the gain setting shown in Table 1. Here, each symbol in Table 1 has a slightly different meaning, i.e., in quantum game, $R_{succ}\left(⋄C\right)$ means that Rice finds the photon after opening the box ⋄C. $B_{succ}\left(⋄B\right)$ means that Bob finds the photon after opening the box ⋄B. ${R/B}_{fail}\left(\cdot \right)$ means that neither Rice or Bob finds the photon and Alice is honest. ${R/B}_{succ}\left(⋄A\right)$ means that neither Rice or Bob finds the photon but Alice is not honest.

#### 3.2.1. The Average Gain of Rice

Denote $G_{Rice}$ as Rice’s average gain. We can easily get $G_{Rice}$ according to Table 1 as follows

(28) $$\begin{array}{ccc} G_{Rice} & = & 2P_{Rice_{1}}+\eta P_{Rice_{2}}-P_{Bob_{1}}-P_{Alice}\\ & = & -\frac{\left(\eta +1\right){\left(\sqrt{\alpha_{1}\omega_{1}}+x\sqrt{\alpha_{2}\omega_{2}}\right)}^{2}}{\omega_{1}+\omega_{2}x}\\ & & +\left(\eta -2\right)\alpha_{2}\gamma +\left(\eta +1\right)\alpha_{2}\beta +\eta \alpha_{1}+\alpha_{2} \end{array}$$

where x=β+γ.

The partial derivative of $G_{Rice}$ with respect to the variable γ is given by

(29) $$\begin{array}{ccc} \frac{\partial G_{Rice}}{\partial \gamma } & = & \frac{1}{{\left(\omega_{1}+\omega_{2}x\right)}^{2}}(-3\omega_{2}^{2}\alpha_{2}x^{2}-6\omega_{1}\omega_{2}\alpha_{2}x\\ & & +\left(\eta -2\right)\omega_{1}^{2}\alpha_{2}+\left(\eta +1\right)\omega_{1}\omega_{2}\alpha_{1}\\ & & -2\omega_{1}\left(\eta +1\right)\sqrt{\alpha_{1}\alpha_{2}\omega_{1}\omega_{2}}) \end{array}$$

Each participant has the perfect knowledge before the game reaching this stage, i.e., each participant is exactly aware of what previous participant has done. Hence, $\omega_{1}+\omega_{2}x\neq 0$. Let $\frac{\partial G_{Rice}}{\partial \gamma }=0$. We get

(30) $$\begin{array}{ccc} x_{1}^{*} & = & -\frac{\sqrt{\omega_{1}\left(\eta +1\right)}\left|\sqrt{\alpha_{1}\omega_{2}}-\sqrt{\alpha_{2}\omega_{1}}\right|}{\omega_{2}\sqrt{3\alpha_{2}}}-\frac{\omega_{1}}{\omega_{2}}\\ x_{2}^{*} & = & \frac{\sqrt{\omega_{1}\left(\eta +1\right)}\left|\sqrt{\alpha_{1}\omega_{2}}-\sqrt{\alpha_{2}\omega_{1}}\right|}{\omega_{2}\sqrt{3\alpha_{2}}}-\frac{\omega_{1}}{\omega_{2}} \end{array}$$

If Alice chooses $\alpha_{1}<\omega_{1}$, the probability that Alice finds the photon will decrease while the probability that Rice or Bob detects the difference between the prepared and the committed states will increase in the verification stage. This means that Alice has no benefit if she chooses $\alpha_{1}<\omega_{1}$. It follows that $\alpha_{1}\geq \omega_{1}$ and $\alpha_{2}\leq \omega_{2}$. Hence, from Equation (30) we get

(31) $$\begin{array}{ccc} x_{1}^{*} & = & -\frac{\sqrt{\omega_{1}\left(\eta +1\right)}\left(\sqrt{\alpha_{1}\omega_{2}}-\sqrt{\alpha_{2}\omega_{1}}\right)}{\sqrt{3}\sqrt{\alpha_{2}}\omega_{2}}-\frac{\omega_{1}}{\omega_{2}}\\ x_{2}^{*} & = & \frac{\sqrt{\omega_{1}\left(\eta +1\right)}\left(\sqrt{\alpha_{1}\omega_{2}}-\sqrt{\alpha_{2}\omega_{1}}\right)}{\sqrt{3}\sqrt{\alpha_{2}}\omega_{2}}-\frac{\omega_{1}}{\omega_{2}} \end{array}$$

From Equation (31), we get $x_{1}^{*}\leq 0$ and $x_{2}^{*}\geq x_{1}^{*}$. From Equations (30) and (31), $G_{Rice}$ is a decreasing function in *x* when $x\leq x_{1}^{*}$ and increasing when $x_{1}^{*}<x\leq x_{2}^{*}$. Moreover, when $x>x_{2}^{*}$, $G_{Rice}$ is a decreasing function in *x*. Since 0≤x≤2, $G_{Rice}$ gets the maximum value at x=0 for $x_{2}^{*}\leq 0$, or gets the maximum value at x=2 for $x_{2}^{*}\geq 2$, or gets the maximum value at $x=x_{2}^{*}$ for $0<x_{2}^{*}<2$.

#### 3.2.2. The Average Gain of Bob

Denote $G_{Bob}$ as the average gain of Bob. From Table 1, we get $G_{Bob}$ as

(32) $$\begin{array}{ccc} G_{Bob} & = & 2P_{Bob_{1}}+P_{Bob_{2}}-P_{Rice_{1}}-P_{Alice}\\ & = & -\frac{2{\left(\sqrt{\alpha_{1}\omega_{1}}+x\sqrt{\alpha_{2}\omega_{2}}\right)}^{2}}{\left(\omega_{1}+\omega_{2}x\right)}+\alpha_{1}+\alpha_{2}\\ & & -\alpha_{2}\beta +2\alpha_{2}\gamma \end{array}$$

where x=β+γ.

The partial derivative of $G_{Bob}$ with respect to β is given by

(33) $$\begin{array}{ccc} \frac{\partial G_{Bob}}{\partial \beta } & = & \frac{1}{{\left(\omega_{1}+\omega_{2}x\right)}^{2}}(-3\omega_{2}^{2}\alpha_{2}x^{2}-6\omega_{1}\omega_{2}\alpha_{2}x\\ & & -\omega_{1}^{2}\alpha_{2}-4\omega_{1}\sqrt{\alpha_{1}\alpha_{2}\omega_{1}\omega_{2}}+2\omega_{1}\omega_{2}\alpha_{1}) \end{array}$$

Similar to Equations (30) and (31), we can get

(34) $$\begin{array}{ccc} x_{3}^{*} & = & -\frac{\sqrt{2\omega_{1}}\left(\sqrt{\alpha_{1}\omega_{2}}-\sqrt{\alpha_{2}\omega_{1}}\right)}{\omega_{2}\sqrt{3\alpha_{2}}}-\frac{\omega_{1}}{\omega_{2}}\\ x_{4}^{*} & = & \frac{\sqrt{2\omega_{1}}\left(\sqrt{\alpha_{1}\omega_{2}}-\sqrt{\alpha_{2}\omega_{1}}\right)}{\omega_{2}\sqrt{3\alpha_{2}}}-\frac{\omega_{1}}{\omega_{2}} \end{array}$$

from $\frac{\partial G_{Bob}}{\partial \beta }=0$.

From Equation (34), we obtain $x_{3}^{*}\leq 0$ and $x_{4}^{*}\geq x_{3}^{*}$. From Equations (33) and (34), $G_{Bob}$ decreases with $x\leq x_{3}^{*}$ and increases with $x_{3}^{*}<x\leq x_{4}^{*}$. Moreover, $G_{Bob}$ decreases with $x>x_{4}^{*}$. Since 0≤x≤2, $G_{Bob}$ gets the maximum value at x=0 for for $x_{4}^{*}\leq 0$, or gets the maximum value at x=2 for $x_{4}^{*}\geq 2$, or gets the maximum value at $x=x_{4}^{*}$ for $0<x_{4}^{*}<2$. From η≥1, we get $x_{2}^{*}\geq x_{4}^{*}$.

Similarly, we can get the detailed analysis of Rice, shown in Appendix F. The values of $\beta^{*}$ and $\gamma^{*}$ which depend on $x_{i}^{*}$ in the first quantum game such that $G_{Bob}$ achieves the maximum are given in Table 7.

#### 3.2.3. The Average Gain of Alice

Denote $G_{Alice}$ as the average gain of Alice. From Table 1, it is easy to evaluate $G_{Alice}$ as follows

(35) $$\begin{array}{ccc} G_{Alice} & = & 2P_{Alice}-\left(\eta +1\right)P_{Rice_{2}}-P_{Bob_{1}}-P_{Rice_{1}}\\ & = & \left(\eta +3\right)\frac{{\left(\sqrt{\alpha_{1}\omega_{1}}+x\sqrt{\alpha_{2}\omega_{2}}\right)}^{2}}{\omega_{1}+\omega_{2}x}\\ & & -\eta \alpha_{2}x-1-\eta \alpha_{1} \end{array}$$

where x=β+γ.

From Table 7, we discuss Alice’s gain in five cases. Here, we only discuss one of them. Other cases are shown in Appendix G.

If $x_{2}^{*}\leq 0$, i.e., $\sqrt{\frac{\alpha_{1}\omega_{1}\left(\eta +1\right)}{3\alpha_{2}\omega_{2}}}-\frac{\omega_{1}}{\omega_{2}}\left(\sqrt{\frac{\eta +1}{3}}+1\right)\leq 0$, we get that

(36) $$\begin{array}{c} \omega_{1}\leq \alpha_{1}\leq \frac{\frac{\omega_{1}}{\omega_{2}}{\left(\sqrt{\frac{\eta +1}{3}}+1\right)}^{2}}{\frac{\omega_{1}}{\omega_{2}}{\left(\sqrt{\frac{\eta +1}{3}}+1\right)}^{2}+\frac{2}{3}\left(\eta +1\right)} \end{array}$$

Denote

$$\begin{array}{c} D=\frac{\frac{\omega_{1}}{\omega_{2}}{\left(\sqrt{\frac{\eta +1}{3}}+1\right)}^{2}}{\frac{\omega_{1}}{\omega_{2}}{\left(\sqrt{\frac{\eta +1}{3}}+1\right)}^{2}+\frac{2}{3}\left(\eta +1\right)} \end{array}$$

It follows that $\omega_{1}\leq \alpha_{1}\leq D$. In this case, we get β=γ=0. From Equation (36), we get that the average gain of Alice is given by

(37) $$\begin{array}{c} G_{Alice}=3\alpha_{1}-1 \end{array}$$

Note that $\frac{dG_{Alice}}{d\alpha_{1}}=3$ from Equation (37). $G_{Alice}$ increases with $\alpha_{1}$ when $\omega_{1}\leq \alpha_{1}\leq D$. So, $G_{Alice}$ gets the maximum value at $\alpha_{1}=D$, which is denoted by $G_{Alice_{1}}$ given by

(38) $$\begin{array}{c} G_{Alice_{1}}=3D-1 \end{array}$$

where the corresponding point is denoted by $p_{1}$.

By using the same method for other cases (see Appendix G), we get Table 8.

#### 3.2.4. Quantum Fair Game

In this subsection, we prove the proposed quantum game is fair. From Equations (29) and (32) and Table 8 we get that

(39) $$\begin{array}{ccc} G_{Alice} & = & \max\{G_{Alice_{1}},G_{Alice_{2}},G_{Alice_{3}},G_{Alice_{4}},G_{Alice_{5}}\}\\ G_{Bob} & = & -\frac{2{\left(\sqrt{\alpha_{1}\omega_{1}}+x\sqrt{\alpha_{2}\omega_{2}}\right)}^{2}}{\omega_{1}+\omega_{2}x}\\ & & +2\alpha_{2}\gamma -\alpha_{2}\beta -\alpha_{2}+1\\ G_{Rice} & = & -\frac{\left(\eta +1\right){\left(\sqrt{\alpha_{1}\omega_{1}}+x\sqrt{\alpha_{2}\omega_{2}}\right)}^{2}}{\omega_{1}+\omega_{2}x}\\ & & +\left(\eta -2\right)\alpha_{2}\gamma +\eta \alpha_{2}\beta +\eta \alpha_{1}+2\alpha_{2} \end{array}$$

**Figure 7:**
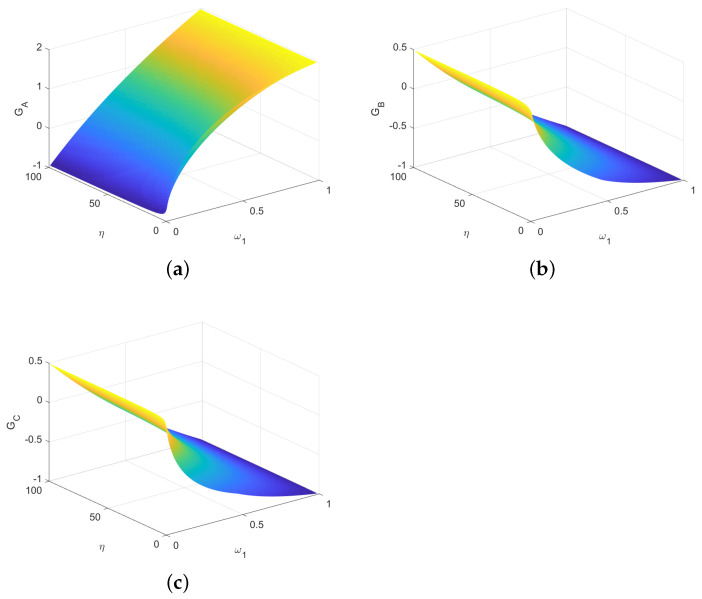
The average gains of three parties depending on η and $\omega_{1}$. (**a**) The average gain of Alice. (**b**) The average gain of Bob. (**c**) The average gain of Rice.

If $G_{Alice_{1}}=\max\{G_{Alice_{1}}$,$G_{Alice_{2}}$,$G_{Alice_{3}},G_{Alice_{4}},G_{Alice_{5}}\}$, from Table 8 we obtain that $\alpha_{1}=p_{1}$, and β=γ=0 from Table 7. The average gains of three players are evaluated using η and $\omega_{1}$, as shown in Figure 7. Here, for each η≥1, the gains of Alice, Bob and Rice can tend to zero by changing $\omega_{1}$.

**Figure 8:**
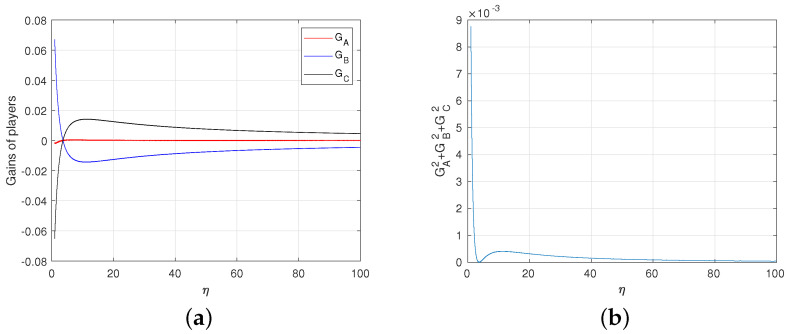
(**a**) The average gains of Alice, Bob and Rice depending on η. For each η, the value of $\omega_{1}$ is equal to the value that minimizes the deviation of the game from fair game. (**b**) Degree of deviation. Here, we express the degree of deviation from the fair game as the sum of the squares of each player’s average gain.

From Figure 8a,b, we get that the degree of deviation from the fair game is very small even if the game is not completely fair. The game converges to the fair game when η→∞. To sum up, we get the following theorem.

Result 3 The first quantum game $G_{1}$ is asymptotically fair.

**Proof.**  Note that the first quantum game $G_{1}$ is zero-sum, i.e., the summation of the average gains of all players is zero. From Equation (36), Alice will make $|\langle \psi_{1}|\psi_{C}\rangle |=0$ when η→∞, i.e., $\alpha_{1}=\omega_{1}$, in order to maximize her own gain, while Bob and Rice will choose β=γ=0 accordingly. Hence, we get three gains as follows

(40) $$\begin{array}{ccc} G_{Alice} & = & 2\omega_{1}-2\omega_{2}\\ G_{Bob} & = & \omega_{2}-\omega_{1}\\ G_{Rice} & = & \omega_{2}-\omega_{1} \end{array}$$

Combining with the assumption of $\omega_{1}+2\omega_{2}=1$, we get $\omega_{2}=\omega_{1}=\frac{1}{3}$ from $G_{Alice}=G_{Bob}=G_{Rice}=0$. Thus, when η→∞, the quantum game $G_{1}$ converges a fair game, i.e., asymptotically fair. This completes the proof. □

### 3.3. The Second Tripartite Quantum Game

In this section, we give the quantum realization of the second game $G_{2}$. According to Table 5, similar to the first quantum game, the gain of Rice is given by

(41) $$\begin{array}{ccc} G_{Rice} & = & -2\frac{{\left(\sqrt{\alpha_{1}\omega_{1}}+x\sqrt{\alpha_{2}\omega_{2}}\right)}^{2}}{\omega_{1}+\omega_{2}x}\\ & & +\left(\mu -1\right)\alpha_{2}\left(1-\gamma \right)+\alpha_{1}+2\alpha_{2}\beta \end{array}$$

where x=β+γ. The detailed maximizing $G_{Rice}$ is shown in Appendix H.

Similarly, the gain of Bob is given by

(42) $$\begin{array}{ccc} G_{Bob} & = & -2\frac{{\left(\sqrt{\alpha_{1}\omega_{1}}+x\sqrt{\alpha_{2}\omega_{2}}\right)}^{2}}{\left(\omega_{1}+\omega_{2}x\right)}+\alpha_{1}\\ & & +\alpha_{2}-\alpha_{2}\beta +2\alpha_{2}\gamma \end{array}$$

Its maximization is shown in Appendix I.

The gain of Alice is given by

(43) $$\begin{array}{ccc} G_{Alice} & = & \frac{4{\left(\sqrt{\alpha_{1}\omega_{1}}+x\sqrt{\alpha_{2}\omega_{2}}\right)}^{2}}{\omega_{1}+\omega_{2}x}-\mu \alpha_{2}\\ & & +\left(\mu -3\right)\alpha_{2}\gamma -2\alpha_{1}-\alpha_{2}\beta \end{array}$$

Its maximization is shown in Appendix J.

**Figure 9:**
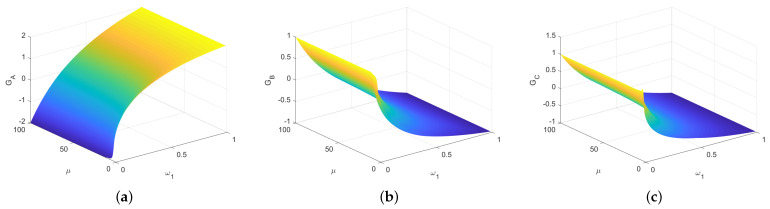
The average gains of three parties depending on μ and $\omega_{1}$.

**Figure 10:**
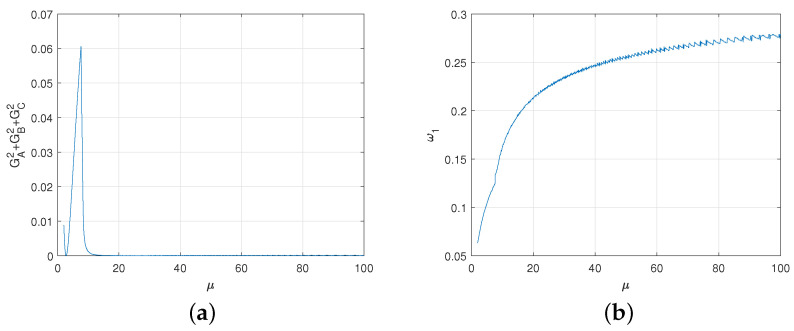
(**a**) For each μ, the degree to which the game deviates from the fair game. Here, we express the degree of deviation from the game as the sum of the squares of each player’s average gain. (**b**) The relationship between the appropriate $\omega_{1}$ and μ.

The numeric evaluations of average gains are shown in Figure 9 in terms of μ and $\omega_{1}$. For each μ, we can make the gains of Alice, Bob and Rice equal to zero as much as possible by adjusting $\omega_{1}$. The deviation degree is shown in Figure 10a. The relationship between the appropriate $\omega_{1}$ and μ is shown in Figure 10b. From Figure 10a,b, the degree of deviation from the fair game is very small even if the game is not completely fair. The game converges to the fair game when μ→∞. To sum up, we get the following result.

Result 4 The second quantum game $G_{2}$ is asymptotically fair.

**Proof.**  Note that the second quantum game $G_{2}$ is zero-sum, i.e., the summation of the average gains of all players is zero. From Equation (46), Alice will make $\alpha_{2}=0$ when μ→∞, i.e., $\alpha_{1}=1$, in order to maximize her own gain, while Bob and Rice will choose β=γ=1 accordingly. Hence, we get three gains as follows

(44) $$\begin{array}{ccc} G_{Alice} & = & 4\omega_{1}-2\\ G_{Bob} & = & 1-2\omega_{1}\\ G_{Rice} & = & 1-2\omega_{1} \end{array}$$

We get $\omega_{1}=\frac{1}{2}$ and $\omega_{2}=\frac{1}{4}$ from $G_{Alice}=G_{Bob}=G_{Rice}=0$. Thus, when μ→∞, the quantum game $G_{2}$ converges a fair game, i.e., asymptotically fair. This completes the proof. □

## 4. Quantum Game with Noises

In this section, we consider quantum games with noises. One is from the experimental measurement. The other is from the preparation noise of resource state.

### 4.1. Experimental Measurement Error

In the case of measurement error, from Equation (20), we can get that the probability of Rice opens box ⋄C and finds the photon becomes

(45) $$\begin{array}{c} P_{Rice_{1}}={\left|\langle c|\psi_{0}\rangle \right|}^{2}+\varepsilon =\alpha_{2}\left(1-\gamma \right)+\varepsilon \end{array}$$

where ε is measurement error which may be very small.

From Equation (20), we get the probability that Bob opens box ⋄B and finds the photon is given by

(46) $$\begin{array}{c} P_{Bob_{1}}={\left|\langle b|\psi_{0}\rangle \right|}^{2}+\varepsilon =\alpha_{2}\left(1-\beta \right)+\varepsilon \end{array}$$

Rice or Bob makes a projection measurement on $|\psi_{C}\rangle$ for the verification the final state with success probability

(47) $$\begin{array}{c} |\langle \psi_{1}|\psi_{C}\rangle {|}^{2}=\frac{\left(\sqrt{\alpha_{1}\omega_{1}}+x\sqrt{\alpha_{2}\omega_{2}}\right)}{\left(\alpha_{1}+\alpha_{2}x\right)\left(\omega_{1}+\omega_{2}x\right)}+\varepsilon \end{array}$$

where x=β+γ.

$P_{Alice}$ is the probability that neither Rice nor Bob finds the photon and Alice did prepare the photon in the committed state, is given by

(48) $$\begin{array}{ccc} P_{Alice} & = & \left(1-P_{Rice_{1}}-P_{Bob_{1}}\right){\left|\langle \psi_{1}|\psi_{C}\rangle \right|}^{2}\\ & = & \frac{{\left(\sqrt{\alpha_{1}\omega_{1}}+x\sqrt{\alpha_{2}\omega_{2}}\right)}^{2}}{\omega_{1}+\omega_{2}x}-2\varepsilon \lambda_{4}\\ & & +\varepsilon \lambda_{3}-2\varepsilon^{2} \end{array}$$

where $\lambda_{i}$ are given by

$$\begin{array}{ccc} \lambda_{3} & = & \alpha_{1}+\alpha_{2}x\leq 1\\ \lambda_{4} & = & \frac{{\left(\sqrt{\alpha_{1}\omega_{1}}+x\sqrt{\alpha_{2}\omega_{2}}\right)}^{2}}{\left(\omega_{1}+\omega_{2}x\right)\left(\alpha_{1}+\alpha_{2}x\right)}\leq 1 \end{array}$$

Since $\lambda_{3}\leq 1$ and $\lambda_{4}\leq 1$, and ε is very small, denotes $O\left(\varepsilon \right)=-2\varepsilon \lambda_{4}+\varepsilon \lambda_{3}-2\varepsilon^{2}$, which can be treated as measurement error. Thus Equation (49) can be rewritten as

(49) $$\begin{array}{c} P_{Alice}=\frac{{\left(\sqrt{\alpha_{1}\omega_{1}}+x\sqrt{\alpha_{2}\omega_{2}}\right)}^{2}}{\omega_{1}+\omega_{2}x}+O\left(\varepsilon \right) \end{array}$$

The probability that neither Rice nor Bob finds the photon but Rice or Bob detects the forage preparation of Alice is denoted by $P_{Rice_{2}}$ or $P_{Bob_{2}}$ which given by

(50) $$\begin{array}{ccc} P_{Rice_{2}} & = & \left(1-P_{Rice_{1}}-P_{Bob_{1}}\right)\left(1-\right|\langle \psi_{1}|\psi_{C}\rangle {|}^{2})\\ & = & \frac{-{\left(\sqrt{\alpha_{1}\omega_{1}}+x\sqrt{\alpha_{2}\omega_{2}}\right)}^{2}}{\omega_{1}+\omega_{2}x}+\alpha_{1}+\alpha_{2}x-2\varepsilon -O\left(\varepsilon \right)\\ & = & \frac{-{\left(\sqrt{\alpha_{1}\omega_{1}}+x\sqrt{\alpha_{2}\omega_{2}}\right)}^{2}}{\omega_{1}+\omega_{2}x}+\alpha_{1}+\alpha_{2}x+O\left(\varepsilon \right) \end{array}$$

where $P_{Rice_{2}}=P_{Bob_{2}}$ from the winning rule W4.

From Equations (21) and (22), Equations (25)–(27), Equations (45)–(47) and Equations (49) and (50), we get the present quantum games are also asymptotically fair if the measurement error is small enough.

### 4.2. White Noises

In this subsection, we consider that Alice prepares a noisy photon in the state

(51) $$\begin{array}{c} \rho_{0}=v|\psi \rangle \langle \psi |+\frac{1-v}{3}I \end{array}$$

where I=|a〉〈a|+|b〉〈b|+|c〉〈c| denotes the identity operator, |ψ〉 is given in Equation (16), and v∈[0,1]. After Bob’s and Rice’s splitting operation, the state of the photon becomes

(52) $$\begin{array}{ccc} \rho_{1} & = & v|\psi_{0}\rangle \langle \psi_{0}|+\frac{1-v}{3}(|a\rangle \langle a|+(\sqrt{1-\beta }|b\rangle \\ & & +\sqrt{\beta }|b^{\prime }\rangle )(\sqrt{1-\beta }\langle b|+\sqrt{\beta }\langle b^{\prime }|)+(\sqrt{1-\gamma }|c\rangle \\ & & +\sqrt{\gamma }|c^{\prime }\rangle )(\sqrt{1-\gamma }\langle c|+\sqrt{\gamma }\langle c^{\prime }|)) \end{array}$$

where $|\psi_{0}\rangle$ is given by

(53) $$\begin{array}{ccc} |\psi_{0}\rangle & = & \sqrt{\alpha_{1}}|a\rangle +\sqrt{\alpha_{2}\left(1-\beta \right)}|b\rangle +\sqrt{\alpha_{2}\beta }|b^{\prime }\rangle \\ & & +\sqrt{\alpha_{2}\left(1-\gamma \right)|c\rangle }+\sqrt{\alpha_{2}\gamma }|c^{\prime }\rangle \end{array}$$

Denote $P_{Rice_{1}}$ as the probability that Rice finds the photon after opening the box ⋄C. From Equation (53) it is given by

(54) $$\begin{array}{c} P_{Rice_{1}}=v\alpha_{2}\left(1-\gamma \right)+\frac{1-v}{3}\left(1-\gamma \right) \end{array}$$

Denote $P_{Bob_{1}}$ as the probability that Bob finds the photon after opening the box ⋄B. From Equation (53) it is given by

(55) $$\begin{array}{c} P_{Bob_{1}}=v\alpha_{2}\left(1-\beta \right)+\frac{1-v}{3}\left(1-\beta \right) \end{array}$$

For the case that neither Bob nor Rice detects the photon, the density operator for the photon is given by

(56) $$\begin{array}{ccc} \rho_{2} & = & \frac{v}{1-P_{Bob_{1}}-P_{Rice_{1}}}\left[\begin{array}{ccccc} \alpha_{1} & \sqrt{\alpha_{1}\alpha_{2}\left(1-\beta \right)} & \sqrt{\alpha_{1}\alpha_{2}\beta } & \sqrt{\alpha_{1}\alpha_{2}\left(1-\gamma \right)} & \sqrt{\alpha_{1}\alpha_{2}\gamma }\\ \sqrt{\alpha_{1}\alpha_{2}\left(1-\beta \right)} & 0 & \alpha_{2}\sqrt{\beta (1-\beta )} & \alpha_{2}\sqrt{\left(1-\beta )(1-\gamma \right)} & \alpha_{2}\sqrt{(1-\beta )\gamma }\\ \sqrt{\alpha_{1}\alpha_{2}\beta } & \alpha_{2}\sqrt{\beta (1-\beta )} & \alpha_{2}\beta & \alpha_{2}\sqrt{\beta (1-\gamma )} & \alpha_{2}\sqrt{\beta \gamma }\\ \sqrt{\alpha_{1}\alpha_{2}\left(1-\gamma \right)} & \alpha_{2}\sqrt{\left(1-\beta )(1-\gamma \right)} & \alpha_{2}\sqrt{\beta (1-\gamma )} & 0 & \alpha_{2}\sqrt{\gamma (1-\gamma )}\\ \sqrt{\alpha_{1}\alpha_{2}\gamma } & \alpha_{2}\sqrt{(1-\beta )\gamma } & \alpha_{2}\sqrt{\beta \gamma } & \alpha_{2}\sqrt{\gamma (1-\gamma )} & \alpha_{2}\gamma \end{array}\right]\\ & & +\frac{1-v}{3(1-P_{Bob_{1}}-P_{Rice_{1}})}\left[\begin{array}{ccccc} 1 & 0 & 0 & 0 & 0\\ 0 & 0 & \sqrt{\beta (1-\beta )} & 0 & 0\\ 0 & \sqrt{\beta (1-\beta )} & \beta & 0 & 0\\ 0 & 0 & 0 & 0 & \sqrt{\gamma (1-\gamma )}\\ 0 & 0 & 0 & \sqrt{\gamma (1-\gamma )} & 1-\gamma \end{array}\right] \end{array}$$

Now, Rice or Bob makes a projection measurement with positive operator $\{|\psi_{C}\rangle \langle \psi_{C}\left|,I-\right|\psi_{C}\rangle \langle \psi_{C}|\}$ on the photon for verifying the committed state of Alice with success probability

(57) $$\begin{array}{ccc} \langle \psi_{C}|\rho_{2}|\psi_{C}\rangle & = & \frac{v{\left(\sqrt{\alpha_{1}\omega_{1}}+x\sqrt{\alpha_{2}\omega_{2}}\right)}^{2}}{\left(\omega_{1}+\omega_{2}x\right)\left(1-P_{Bob_{1}}-P_{Rice_{1}}\right)}\\ & & +\frac{1-v}{3}\frac{\omega_{1}+\omega_{2}\beta^{2}+\omega_{2}\gamma^{2}}{\left(\omega_{1}+\omega_{2}x\right)\left(1-P_{Bob_{1}}-P_{Rice_{1}}\right)} \end{array}$$

Denote $P_{Alice}$ as the probability that neither Rice nor Bob finds the photon, and Alice did prepare the photon in the committed state. It is easy to obtain that

(58) $$\begin{array}{ccc} P_{Alice} & = & \left(1-P_{Rice_{1}}-P_{Bob_{1}}\right)\langle \psi_{C}|\rho_{2}|\psi_{C}\rangle \\ & = & \frac{v{\left(\sqrt{\alpha_{1}\omega_{1}}+x\sqrt{\alpha_{2}\omega_{2}}\right)}^{2}}{\omega_{1}+\omega_{2}x}\\ & & +\frac{\left(1-v\right)\left(\omega_{1}+\omega_{2}\beta^{2}+\omega_{2}\gamma^{2}\right)}{3(\omega_{1}+\omega_{2}x)} \end{array}$$

Denote $P_{Rice_{2}}$ or $P_{Bob_{2}}$ as the probability that neither Rice nor Bob finds the photon but Rice or Bob detects the forage preparation. We obtain that

(59) $$\begin{array}{ccc} P_{Rice_{2}} & = & \left(1-P_{Rice_{1}}-P_{Bob_{1}}\right)(1-\langle \psi_{C}|\rho_{2}|\psi_{C}\rangle )\\ & = & 1-P_{Rice_{1}}-P_{Bob_{1}}-P_{Alice} \end{array}$$

where $P_{Rice_{2}}=P_{Bob_{2}}$ from the winning rule W4.

Take the first quantum game as an example. The Rice’s average gain $G_{Rice}$ is given by

(60) $$\begin{array}{ccc} G_{Rice} & = & 2P_{Rice_{1}}+\eta P_{Rice_{2}}-P_{Bob_{1}}-P_{Alice}\\ & = & \left(v\alpha_{2}+\frac{1-v}{3}\right)\left(2-\eta \right)\left(1-\gamma \right)\\ & & -\left(v\alpha_{2}+\frac{1-v}{3}\right)\left(\eta +1\right)\left(1-\beta \right)+\eta \\ & & -\left(\eta +1\right)(v\frac{{\left(\sqrt{\alpha_{1}\omega_{1}}+x\sqrt{\alpha_{2}\omega_{2}}\right)}^{2}}{\omega_{1}+\omega_{2}x}\\ & & +\frac{1-v}{3}\frac{\omega_{1}+\omega_{2}\beta^{2}+\omega_{2}\gamma^{2}}{\omega_{1}+\omega_{2}x}) \end{array}$$

The partial derivative of $G_{Rice}$ with respect to γ is

(61) $$\begin{array}{ccc} \frac{\partial G_{Rice}}{\partial \gamma } & = & \frac{v}{{\left(\omega_{1}+\omega_{2}x\right)}^{2}}(-3\omega_{2}^{2}\alpha_{2}x^{2}-6\omega_{1}\omega_{2}\alpha_{2}x\\ & & +\left(\eta -2\right)\omega_{1}^{2}\alpha_{2}+\left(\eta +1\right)\omega_{1}\omega_{2}\alpha_{1}\\ & & -2\omega_{1}\left(\eta +1\right)\sqrt{\alpha_{1}\alpha_{2}\omega_{1}\omega_{2}})+\frac{1-v}{3}\epsilon \end{array}$$

where $\epsilon =-\frac{\left(\eta +1\right)\left(2\omega_{1}\omega_{2}\gamma +2\omega_{2}^{2}\beta \gamma +\omega_{2}^{2}\gamma^{2}-\omega_{1}\omega_{2}-\omega_{2}^{2}\beta^{2}\right)}{{\left(\omega_{1}+\omega_{2}x\right)}^{2}}+\eta -2$.

Assume that *v* is very close to one, i.e., $\frac{1-v}{3}\approx 0$. It follows that ϵ is bounded. Thus Equation (62) can be rewritten as

(62) $$\begin{array}{ccc} \frac{\partial G_{Rice}}{\partial \gamma } & = & \frac{v}{{\left(\omega_{1}+\omega_{2}x\right)}^{2}}(-3\omega_{2}^{2}\alpha_{2}x^{2}-6\omega_{1}\omega_{2}\alpha_{2}x\\ & & +\left(\eta -2\right)\omega_{1}^{2}\alpha_{2}+\left(\eta +1\right)\omega_{1}\omega_{2}\alpha_{1}\\ & & -2\omega_{1}\left(\eta +1\right)\sqrt{\alpha_{1}\alpha_{2}\omega_{1}\omega_{2}})+O\left(\epsilon \right) \end{array}$$

Similarly, we get

(63) $$\begin{array}{ccc} G_{Bob} & = & \left(v\alpha_{2}+\frac{1-v}{3}\right)\left(1-\beta \right)\\ & & -2\left(v\alpha_{2}+\frac{1-v}{3}\right)\left(1-\gamma \right)+1\\ & & -\frac{2v{\left(\sqrt{\alpha_{1}\omega_{1}}+x\sqrt{\alpha_{2}\omega_{2}}\right)}^{2}}{\omega_{1}+\omega_{2}x}\\ & & -\frac{2\left(1-v\right)\left(\omega_{1}+\omega_{2}\beta^{2}+\omega_{2}\gamma^{2}\right)}{3(\omega_{1}+\omega_{2}x)} \end{array}$$

The partial derivative of $G_{Bob}$ with respect to β is given by

(64) $$\begin{array}{ccc} \frac{\partial G_{Bob}}{\partial \beta } & = & \frac{v}{{\left(\omega_{1}+\omega_{2}x\right)}^{2}}(-3\omega_{2}^{2}\alpha_{2}x^{2}-6\omega_{1}\omega_{2}\alpha_{2}x\\ & & -\omega_{1}^{2}\alpha_{2}-4\omega_{1}\sqrt{\alpha_{1}\alpha_{2}\omega_{1}\omega_{2}}+2\omega_{1}\omega_{2}\alpha_{1})\\ & & +\frac{1-v}{3}\epsilon \\ & = & \frac{v}{{\left(\omega_{1}+\omega_{2}x\right)}^{2}}(-3\omega_{2}^{2}\alpha_{2}x^{2}-6\omega_{1}\omega_{2}\alpha_{2}x\\ & & -\omega_{1}^{2}\alpha_{2}-4\omega_{1}\sqrt{\alpha_{1}\alpha_{2}\omega_{1}\omega_{2}}\\ & & +2\omega_{1}\omega_{2}\alpha_{1})+O\left(\epsilon \right) \end{array}$$

where $\epsilon =-\frac{4\omega_{1}\omega_{2}\gamma +4\omega_{2}^{2}\beta \gamma +2\omega_{2}^{2}\gamma^{2}-2\omega_{1}\omega_{2}-2\omega_{2}^{2}\beta^{2}}{{\left(\omega_{1}+\omega_{2}x\right)}^{2}}-1$.

The Alice’s average gain $G_{Alice}$ is given by

(65) $$\begin{array}{ccc} G_{Alice} & = & \frac{v\left(\eta +3\right){\left(\sqrt{\alpha_{1}\omega_{1}}+x\sqrt{\alpha_{2}\omega_{2}}\right)}^{2}}{\omega_{1}+\omega_{2}x}\\ & & +\eta v\alpha_{2}\left(2-x\right)-\eta -1\\ & & +\frac{1-v}{3}(\frac{\left(\eta +3\right)\left(\omega_{1}+\omega_{2}\beta^{2}+\omega_{2}\gamma^{2}\right)}{\omega_{1}+\omega_{2}x}\\ & & +\eta (2-x)) \end{array}$$

(66) $$\begin{array}{ccc} & & \;\;\;\;\;\;\;\;=\frac{v\left(\eta +3\right){\left(\sqrt{\alpha_{1}\omega_{1}}+x\sqrt{\alpha_{2}\omega_{2}}\right)}^{2}}{\omega_{1}+\omega_{2}x}+v\eta -\eta \\ & & \;\;\;\;\;\;\;\;-v\eta \alpha_{2}x-v\eta \alpha_{1}-1+O\left(\varepsilon \right) \end{array}$$

From Equations (30), (33), (36), (63), (65) and (66), we get that the first quantum game is asymptotically fair if white noisy is small enough, i.e., *v* is close to 1.

## 5. Conclusions

It has shown that two present quantum games are asymptotically fair. Interestingly, these games can be easily changed to biased versions from Figure 5 and Figure 8, by choosing different η and $\omega_{1}$. These kind of schemes may be applicable in gambling theory. Similar to bipartite scheme [27], a proof-of-principle optical demonstration may be followed for each scheme.

In this paper, we present one tripartite zero-sum game with different settings. This game is unfair if all parties use of classical resources. Interestingly, this can be resolved by using only pure state in similar quantum games. Comparing with the classical games, the present quantum games provide asymptotically fair. Moreover, these quantum games are robust against the measurement errors and preparation noises. This kind of protocols provide interesting features of pure state in resolving specific tasks. The present examples may be extended for multipartite games in theory. Unfortunately, these extensions should be nontrivial because of high complexity depending on lots of free parameters.

## Figures and Tables

**Figure A1:**
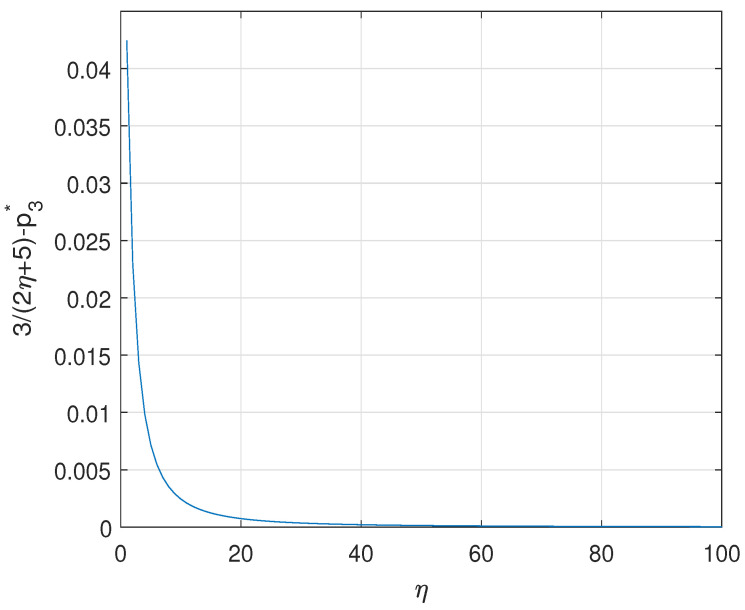
The values of $\frac{3}{2\eta +5}-p_{3}^{*}$ depending on η.

**Table 1:** The gain settings of players in the first classical game $G_{1}$. Here, $g_{c(b,a)}$ denotes the gain of Rice (or Bob, or Alice). $R_{succ}\left(⋄C\right)$ means that Rice finds the ball after opening box ⋄C. $B_{succ}\left(⋄B\right)$ means that Bob finds the ball after opening box ⋄B. ${R/B}_{fail}\left(\cdot \right)$ means that neither Rice or Bob finds the ball. ${R/B}_{succ}\left(⋄A\right)$ means that either Rice or Bob successes by opening box ⋄A. η≥1 in the first classical game.

Cases∖Gains	$g_{c}$	$g_{b}$	$g_{a}$
$R_{succ}\left(⋄C\right)$	2	−1	−1
$B_{succ}\left(⋄B\right)$	−1	2	−1
$R/B_{fail}\left(\cdot \right)$	−1	−1	2
$R/B_{succ}\left(⋄A\right)$	η	1	−η−1

**Table 2:** The values of $P_{⋄B}$ and $P_{⋄C}$. $P_{⋄B(⋄C)}$ depends on the different cases in the game, where $X_{1}=\frac{\eta +8-\sqrt{\eta^{2}+4\eta +52}}{2\eta +2}$. Here, $\Delta_{i}$ denotes the intervals given by $\Delta_{1}=\left[0,\frac{3}{2\eta +5}\right)$, $\Delta_{2}=\left[\frac{3}{2\eta +5},X_{1}\right)$, $\Delta_{3}=\left[X_{1},\frac{3}{\eta +1}\right]$ and $\Delta_{4}=\left(\frac{3}{\eta +1},1\right]$.

η		1≤η≤2	η>2		
$P_{1}$	$P_{1}\in \Delta_{1}$	$P_{1}\in \Delta_{2}$	$P_{1}\in \Delta_{2}$	$P_{1}\in \Delta_{3}$	$P_{1}\in \Delta_{4}$
$P_{⋄B}$	1	$X_{0}$	$\frac{3P_{2}-\left(\eta +1\right)P_{1}P_{2}}{P_{1}\left(\eta +1\right)\left(1-P_{2}\right)}$	$X_{0}$	[0,1]
$P_{⋄C}$	1	0	1	0	0

**Table 3:** $G_{Alice}^{m}$ denotes the maximum of $G_{Alice}$, and $P_{\max}$ denotes the corresponding point of $P_{1}$.

$P_{1}$	$0\leq P_{1}<\frac{3}{2\eta +5}$	$\frac{3}{2\eta +5}\leq P_{1}\leq 1$
$P_{\max}$	$\frac{3}{2\eta +5}-\epsilon$	$\frac{3}{2\eta +5}$
$G_{Alice}^{m}$	$\frac{-\eta^{2}+4\eta +23}{{\left(2\eta +5\right)}^{2}}+O\left(\epsilon \right)$	$\frac{\eta +1}{2\eta +5}$

**Table 4:** The average gains of players in the first classical game $G_{1}$.

Gain∖η	1≤η<2	η≥2
$G_{Alice}$	$\frac{-\eta^{2}+4\eta +23}{{\left(2\eta +5\right)}^{2}}+O\left(\epsilon \right)$	$\frac{1+\eta }{2\eta +5}$
$G_{Bob}$	$\frac{-\eta^{2}-5\eta -13}{{\left(2\eta +5\right)}^{2}}+O(\epsilon )$	$\frac{1-2\eta }{2\eta +5}$
$G_{Rice}$	$\frac{\eta -2}{2\eta +5}+O(\epsilon )$	$\frac{\eta -2}{2\eta +5}$

**Table 5:** The gain settings of the second game $G_{2}$. Here, $g_{c(b,a)}$ denotes gain of Rice (or Bob, or Alice). We assume that μ≥2 in this second game.

Cases∖Gains	$g_{c}$	$g_{b}$	$g_{a}$
$R_{succ}\left(⋄C\right)$	μ	−1	1 − μ
$B_{succ}\left(⋄B\right)$	−1	2	−1
$R/B_{fail}\left(\cdot \right)$	−1	−1	2
$R/B_{succ}\left(⋄A\right)$	1	1	−2

**Table 6:** The average gains of players in the second classical tripartite game $G_{2}$.

Gain∖μ	$2\leq \mu \leq \frac{41\;+\;\sqrt{1345}}{14}$	$\mu >\frac{41\;+\;\sqrt{1345}}{14}$
$G_{Alice}$	$\frac{54\;-\;14\mu }{49}$	$\frac{6\;-\;2\mu }{\mu \;+\;5}$
$G_{Bob}$	$-\frac{19}{49}$	$\frac{\mu \;-\;3}{\mu \;+\;5}$
$G_{Rice}$	$\frac{2\mu \;-\;5}{7}$	$\frac{\mu \;-\;3}{\mu \;+\;5}$

**Table 7:** The values of $\beta^{*}$ and $\gamma^{*}$ depend on $x_{i}^{*}$ in the first quantum game such that $G_{Rice}$ and $G_{Bob}$ achieve the maximums, where $\beta^{*}$ and $\gamma^{*}$ are the probability of Bob opening box $⋄B^{\prime }$ and Rice opening box $⋄C^{\prime }$ respectively. Here, $x_{1}^{*}$ and $x_{2}^{*}$ are given in Equation (31), and $x_{3}^{*}$ and $x_{4}^{*}$ are given in Equation (34).

Cases	$x_{2}^{*}\leq 0$	$0<x_{2}^{*}\leq 1$	$x_{4}^{*}\leq 1<x_{2}^{*}$	$1<x_{4}^{*}<2$	$x_{4}^{*}\geq 2$
$\beta^{*}$	0	0	0	$x_{4}^{*}-1$	1
$\gamma^{*}$	0	$x_{2}^{*}$	1	1	1

**Table 8:** $G_{Alice}^{m}$ denotes the maximum of $G_{Alice}$ where the corresponding point is denoted by $p_{\max}$.

Cases	$x_{2}^{*}\leq 0$	$0<x_{2}^{*}\leq 1$	$x_{4}^{*}\leq 1<x_{2}^{*}$	$1<x_{4}^{*}<2$	$x_{4}^{*}\geq 2$
$p_{\max}$	$p_{1}$	$p_{2}$	$p_{3}$	$p_{4}$	$p_{5}$
$G_{Alice}^{m}$	$G_{Alice_{1}}$	$G_{Alice_{2}}$	$G_{Alice_{3}}$	$G_{Alice_{4}}$	$G_{Alice_{5}}$

## Data Availability

The data from numeric evaluations can be obtained from authors under proper requirements and goals.

## Appendix A. The Average Gain of Bob in the First Classical Game

In order to calculate Bob’s average gain, we need to consider whether $\frac{\partial G_{Rice}}{\partial P_{⋄C}}$ is greater than zero or not. And the case of $\frac{\partial G_{Rice}}{\partial P_{⋄C}}\leq 0$ has given in Section 2.2.2 The case of $\frac{\partial G_{Rice}}{\partial P_{⋄C}}>0$ is as follows.

For $\frac{\partial G_{Rice}}{\partial P_{⋄C}}>0$, we have $0\leq P_{⋄B}<\frac{3P_{2}-P_{1}P_{2}\left(\eta +1\right)}{P_{1}\left(\eta +1\right)\left(1-P_{2}\right)}$. In this case, $P_{⋄C}=1$. From Equation (8) we get

(A1) $$\begin{array}{cc} G_{Bob} & =P_{⋄B}\left(-3P_{2}^{2}+3P_{2}-2P_{1}+2P_{1}P_{2}\right)\\ \; & +2P_{1}-2P_{1}P_{2}-1 \end{array}$$

The partial derivative of $G_{Bob}$ with respect to $P_{⋄B}$ is given by

(A2) $$\begin{array}{c} \frac{\partial G_{Bob}}{\partial P_{⋄B}}=\frac{\left(1-P_{2}\right)\left(3-7P_{1}\right)}{2} \end{array}$$

Two cases will be considered as follows.

(i) If $0<\frac{3P_{2}-P_{1}P_{2}\left(\eta +1\right)}{P_{1}\left(\eta +1\right)\left(1-P_{2}\right)}\leq 1$, we get $\frac{3}{2\eta +5}\leq P_{1}<\frac{3}{\eta +1}$. In this case, there are two subcases.
  C1 For $\frac{3}{2\eta +5}\leq P_{1}\leq \frac{3}{7}$, we get $\frac{\partial G_{Bob}}{\partial P_{⋄B}}\geq 0$, and $G_{Bob}$ increases with $P_{⋄B}$. So, Bob should make $P_{⋄B}=\frac{3P_{2}-P_{1}P_{2}\left(\eta +1\right)}{P_{1}\left(\eta +1\right)\left(1-P_{2}\right)}$ in order to maximize his gains. From Equation (A1), we obtain
    (A3) $$\begin{array}{c} G_{Bob_{2}}=\frac{3P_{2}\left(3P_{2}-2P_{1}\right)}{\left(\eta +1\right)P_{1}}-3P_{2}^{2}+2P_{1}-1 \end{array}$$
  C2 For $\frac{3}{7}<P_{1}<\frac{3}{\eta +1}$, we get $\frac{\partial G_{Bob}}{\partial P_{⋄B}}<0$. Hence, $G_{Bob}$ decreases with $P_{⋄B}$. Thus, Bob will let $P_{⋄B}=0$ in order to maximize his gains. From Equation (A1), we get
    (A4) $$\begin{array}{c} G_{Bob_{3}}=2P_{1}-2P_{1}P_{2}-1 \end{array}$$
(ii) If $\frac{3P_{2}-P_{1}P_{2}\left(\eta +1\right)}{P_{1}\left(\eta +1\right)\left(1-P_{2}\right)}>1$, we get $P_{1}<\frac{3}{2\eta +5}$. Since η≥1, it follows that $\frac{3}{2\eta +5}\leq \frac{3}{7}$. Note that $\frac{\partial G_{Bob}}{\partial P_{⋄B}}\geq 0$. It implies that $G_{Bob}$ increases with $P_{⋄B}$. Bob will set $P_{⋄B}=1$ to maximize his gains. From Equation (A1), we obtain that
  (A5) $$\begin{array}{cc} G_{Bob_{4}} & =3P_{2}-3P_{2}^{2}-1 \end{array}$$

It is no doubt for Bob to choose the strategy which has higher gains. There are two options for Bob.

**Case 1.**$\frac{3}{2\eta +5}\leq P_{1}\leq \frac{3}{7}$.

In this case, we get from Equations (8) and (A3) that

(A6) $$\begin{array}{cc} G_{Bob_{2}}-G_{Bob_{1}} & =\frac{3P_{2}\left(3P_{2}-2P_{1}-P_{1}P_{2}\left(\eta +1\right)\right)}{P_{1}\left(\eta +1\right)}\\ \; & =\frac{3P_{2}\left(P_{1}^{2}\left(\eta +1\right)-P_{1}\left(\eta +8\right)+3\right)}{2P_{1}\left(\eta +1\right)} \end{array}$$

Suppose that $G_{Bob_{2}}-G_{Bob_{1}}=0$. We get $P_{1}^{2}\left(\eta +1\right)-P_{1}\left(\eta +8\right)+3=0$. The solution of this equation is given by $X_{1}=\frac{\eta +8-\sqrt{\eta^{2}+4\eta +52}}{2\eta +2}$ and $X_{2}=\frac{\eta +8+\sqrt{\eta^{2}+4\eta +52}}{2\eta +2}$. It means that $G_{Bob_{2}}-G_{Bob_{1}}>0$ for $P_{1}\in \left[0,X_{1}\right)$ and $P_{1}\in \left(X_{2},1\right)$, and $G_{Bob_{2}}-G_{Bob_{1}}\leq 0$ for $P_{1}\in \left[X_{1},X_{2}\right]$. Note that

(A7) $$\begin{array}{cc} \frac{3}{7}-X_{2} & =\frac{3}{7}-\frac{\eta +8+\sqrt{\eta^{2}+4\eta +52}}{2\eta +2}\\ \; & =\frac{-\eta -50-7\sqrt{\eta^{2}+4\eta +52}}{14(\eta +1)}\\ \; & <0 \end{array}$$

Moreover, it is easy to prove that

(A8) $$\begin{array}{cc} \frac{3}{7}-X_{1} & =\frac{3}{7}-\frac{\eta +8-\sqrt{\eta^{2}+4\eta +52}}{2\eta +2}\\ \; & =\frac{-\eta -50+7\sqrt{\eta^{2}+4\eta +52}}{14(\eta +1)}\\ \; & >0 \end{array}$$

Similarly, we can get

(A9) $$\begin{array}{cc} \frac{3}{2\eta +5}-X_{1} & =\frac{3}{2\eta +5}-\frac{\eta +8-\sqrt{\eta^{2}+4\eta +52}}{2\eta +2}\\ \; & =\frac{-2\eta^{2}-15\eta -34+\left(2\eta +5\right)\sqrt{\eta^{2}+4\eta +52}}{2(\eta +1)(2\eta +5)} \end{array}$$

From Equation (A9), we get $\frac{3}{2\eta +5}-X_{1}\geq 0$ for 1≤η≤2, and $\frac{3}{2\eta +5}-X_{1}<0$ for η>2.

To sum up we get the following result

- For 1≤η≤2, i.e., $X_{1}\leq \frac{3}{2\eta +5}<\frac{3}{7}<X_{2}$, we get
  (A10) $$\begin{array}{c} P_{⋄B}=X_{0} \end{array}$$
  for $P_{1}\in \left[\frac{3}{2\eta +5},\frac{3}{7}\right]$
- For η>2, i.e., $\frac{3}{2\eta +5}<X_{1}<\frac{3}{7}<X_{2}$, we get
  (A11) $$\begin{array}{c} P_{⋄B}=\left\{\begin{array}{cc} \frac{3P_{2}-\left(\eta +1\right)P_{1}P_{2}}{\left(\eta +1\right)P_{1}\left(1-P_{2}\right)}, & P_{1}\in \left[\frac{3}{2\eta +5},X_{1}\right)\\ X_{0}, & P_{1}\in \left[X_{1},\frac{3}{7}\right] \end{array}\right. \end{array}$$
  where $X_{0}\geq \frac{3P_{2}-P_{1}P_{2}\left(\eta +1\right)}{P_{1}\left(\eta +1\right)\left(1-P_{2}\right)}$.

**Case 2.**$\frac{3}{7}<P_{1}<\frac{3}{\eta +1}$.

In this case, from Equations (8) and (A4) we get

(A12) $$\begin{array}{cc} G_{Bob_{3}}-G_{Bob_{1}} & =-2P_{1}P_{2}<0. \end{array}$$

Thus $P_{⋄B}=X_{0}$ for $\frac{3}{7}<P_{1}<\frac{3}{\eta +1}$, where $X_{0}>\frac{3P_{2}-\left(\eta +1\right)P_{1}P_{2}}{\left(\eta +1\right)P_{1}\left(1-P_{2}\right)}$.

## Appendix B. The Average Gain of Alice in the First Classical Game

For the case of $0\leq P_{1}<\frac{3}{2\eta +5}$, we have given in Section 2.2.3. The remaining three cases are as follows.

**Case 1.** For $\frac{3}{2\eta +5}\leq P_{1}<X_{1}$ and 1≤η≤2, we have $P_{⋄C}=0$ and $P_{⋄B}=X_{0}$. From Equation (9), we get

(A13) $$\begin{array}{c} G_{Alice}=2-P_{1}\left(\eta +3\right) \end{array}$$

So, $G_{Alice}$ takes the maximum value at $P_{1}=\frac{3}{2\eta +5}$, which is then denoted by $G_{Alice_{2}}$ given by

(A14) $$\begin{array}{c} G_{Alice_{2}}=\frac{1+\eta }{2\eta +5} \end{array}$$

**Case 2.** For $\frac{3}{2\eta +5}\leq P_{1}<X_{1}$ and η>2, we have $P_{⋄C}=1$ and $P_{⋄B}=\frac{3P_{2}-\left(\eta +1\right)P_{1}P_{2}}{\left(\eta +1\right)P_{1}\left(1-P_{2}\right)}$. From Equation (9), we get

(A15) $$\begin{array}{cc} G_{Alice}= & 3P_{2}^{2}-3P_{2}+2-P_{1}\left(\eta +3\right)\\ \; & +\frac{3P_{2}\left(P_{1}\left(\eta +3\right)-3P_{2}\right)}{P_{1}\left(\eta +1\right)}\\ = & \frac{3}{4}P_{1}^{2}-\left(\eta +\frac{9}{2}\right)P_{1}-\frac{21P_{1}+30}{4(\eta +1)}\\ \; & -\frac{9}{4P_{1}\left(\eta +1\right)}+\frac{11}{4} \end{array}$$

The partial derivative of $G_{Alice}$ with respect to $P_{1}$ is given by

(A16) $$\begin{array}{c} \frac{\partial G_{Alice}}{\partial P_{1}}=\frac{9}{4P_{1}^{2}\left(\eta +1\right)}-\frac{21}{4(\eta +1)}+\frac{3}{2}P_{1}-\frac{9}{2}-\eta \end{array}$$

By setting $\frac{\partial G_{Alice}}{\partial P_{1}}=0$, we get

(A17) $$\begin{array}{c} 6P_{1}^{3}\left(\eta +1\right)-P_{1}^{2}\left(4\eta^{2}+22\eta +39\right)+9=0 \end{array}$$

Let $P_{1}=y+\frac{4\eta^{2}+22\eta +39}{18(\eta +1)}$. From Equation (A15), we get

(A18) $$\begin{array}{cc} \; & y^{3}-\frac{{\left(4\eta^{2}+22\eta +39\right)}^{2}}{108{\left(\eta +1\right)}^{2}}y\\ \; & +\frac{3}{2(\eta +1)}-\frac{{\left(4\eta^{2}+22\eta +39\right)}^{3}}{2916{\left(\eta +1\right)}^{3}}=0 \end{array}$$

The general solution of this equation is given by

$$\begin{array}{c} \left\{\begin{array}{cc} r & =\frac{{\left(4\eta^{2}+22\eta +39\right)}^{3}}{3^{3}\times 6^{3}\times {\left(\eta +1\right)}^{3}}\\ \theta & =\frac{1}{3}\arccos\left(1-\frac{4374{\left(\eta +1\right)}^{2}}{{\left(4\eta^{2}+22\eta +39\right)}^{3}}\right)\\ y_{1} & =2\sqrt[{3}]{r}\cos\theta \\ y_{2} & =2\sqrt[{3}]{r}\cos\left(\theta +\frac{2}{3}\pi \right)\\ y_{3} & =2\sqrt[{3}]{r}\cos\left(\theta +\frac{4}{3}\pi \right) \end{array}\right. \end{array}$$

By substituting $y_{1}$, $y_{2}$ and $y_{3}$ into $P_{1}=y+\frac{4\eta^{2}+22\eta +39}{18(\eta +1)}$, we obtain that

$$\begin{array}{cc} p_{1}^{*} & =y_{1}+\frac{4\eta^{2}+22\eta +39}{18(\eta +1)}\\ \; & =\frac{4\eta^{2}+22\eta +39}{18(\eta +1)}\left(1+2\cos\theta \right)>1\\ p_{2}^{*} & =y_{2}+\frac{4\eta^{2}+22\eta +39}{18(\eta +1)}\\ \; & =\frac{4\eta^{2}+22\eta +39}{18(\eta +1)}\left(1+2\cos\left(\theta +\frac{2}{3}\pi \right)\right)<0\\ p_{3}^{*} & =y_{3}+\frac{4\eta^{2}+22\eta +39}{18(\eta +1)}\\ \; & =\frac{4\eta^{2}+22\eta +39}{18(\eta +1)}\left(1+2\cos\left(\theta +\frac{4}{3}\pi \right)\right)>0 \end{array}$$

Equation (A17) is a cubic equation about $P_{1}$. It is the most likely to have three roots, i.e., $p_{1}^{*}$, $p_{2}^{*}$, $p_{3}^{*}$ with $p_{2}^{*}<0<p_{3}^{*}<1<p_{1}^{*}$. So, $G_{Alice}$ decreases with $P_{1}$ for $P_{1}\in \left(-\infty ,p_{2}^{*}\right)$ and $P_{1}\in \left(p_{3}^{*},p_{1}^{*}\right)$. $G_{Alice}$ increases with $P_{1}$ for $P_{1}\in \left(p_{2}^{*},p_{3}^{*}\right)$ and $P_{1}\in \left(p_{1}^{*},\infty \right)$. From Figure A1, we get $\frac{3}{2\eta +5}>p_{3}^{*}$. So, $G_{Alice}$ takes the maximum value at $P_{1}=\frac{3}{2\eta +5}$, which is then denoted by $G_{Alice_{3}}$ given by

(A19) $$\begin{array}{c} G_{Alice_{3}}=\frac{\eta +1}{2\eta +5} \end{array}$$

**Case 3.** For $X_{1}\leq P_{1}\leq 1$, we have $P_{⋄C}=0$. From Equation (9), we get

(A20) $$\begin{array}{c} G_{Alice}=2-P_{1}\left(\eta +3\right) \end{array}$$

From Equation (A20) $G_{Alice}$ decreases $P_{1}$. Hence, the maximum value $G_{Alice_{4}}$ of $G_{Alice}$ is given by

(A21) $$\begin{array}{c} G_{Alice_{4}}=2-X_{1}\left(\eta +3\right) \end{array}$$

Note that $G_{Alice_{2}},G_{Alice_{3}}>G_{Alice_{4}}$. Thus, $G_{Alice}$ gets the maximum value $\frac{\eta +1}{2\eta +5}$ at $P_{1}=\frac{3}{2\eta +5}$ for $\frac{3}{2\eta +5}\leq P_{1}\leq 1$.

## Appendix C. The Average Gain of RICE in the Second Classical Game

It is straightforward to calculate the average gain of Rice according to Table 5 as follows

(A22) $$\begin{array}{cc} G_{Rice}= & \mu P_{Rice_{1}}-P_{Bob_{1}}-P_{Alice}+P_{Rice_{2}}\\ = & \mu P_{⋄C}P_{2}-P_{⋄B}P_{⋄C}\left(1-P_{2}\right)\\ \; & +\left(1-P_{⋄C}\right)\left(2P_{1}-1\right)+P_{⋄C}\left(1-P_{2}\right)(1\\ \; & -P_{⋄B})\left(2P_{1}-1\right) \end{array}$$

The partial derivative of $G_{Rice}$ with respect to $P_{⋄C}$ is given by

(A23) $$\begin{array}{cc} \frac{\partial G_{Rice}}{\partial P_{⋄C}}= & \left(\mu +1\right)P_{2}-2P_{1}P_{⋄B}+2P_{1}P_{⋄B}P_{2}-2P_{1}P_{2} \end{array}$$

If $\frac{\partial G_{Rice}}{\partial P_{⋄C}}>0$, i.e., $G_{Rice}$ increases with $P_{⋄C}$, Rice will choose $P_{⋄C}=1$ to maximize her gains. However, if $\frac{\partial G_{Rice}}{\partial P_{⋄C}}\leq 0$, i.e., $G_{Rice}$ decreases with $P_{⋄C}$, Rice will set $P_{⋄C}=0$ in order to maximize her gains.

## Appendix D. The Average Gain of Bob in Classical Second Game

Similar to $G_{Rice}$, we can get Bob’s average gain $G_{Bob}$ as follows

(A24) $$\begin{array}{cc} G_{Bob}= & -P_{Rice_{1}}+2P_{Bob_{1}}-P_{Alice}+P_{Bob_{2}}\\ = & P_{⋄B}P_{⋄C}\left(1-P_{2}\right)\left(3P_{2}-1\right)\\ \; & +P_{⋄C}\left(1-P_{2}\right)\left(1-P_{⋄B}\right)\left(2P_{1}-1\right)\\ \; & +\left(1-P_{⋄C}\right)\left(2P_{1}-1\right)-P_{⋄C}P_{2} \end{array}$$

According to the sign of $\frac{\partial G_{Rice}}{\partial P_{⋄C}}$, we can divide it into two cases.

**Case 1.**$\frac{\partial G_{Rice}}{\partial P_{⋄C}}>0$, i.e., $0\leq P_{⋄B}<\frac{\left(\mu +1\right)P_{2}-2P_{1}P_{2}}{2P_{1}\left(1-P_{2}\right)}$.

In this case, $P_{⋄C}=1$. From Equation (A25) we get

(A25) $$\begin{array}{cc} G_{Bob}= & -P_{2}+P_{⋄B}\left(1-P_{2}\right)\left(3P_{2}-1\right)\\ \; & +\left(1-P_{2}\right)\left(1-P_{⋄B}\right)\left(2P_{1}-1\right) \end{array}$$

The partial derivative of $G_{Bob}$ with respect to $P_{⋄B}$ is given by

(A26) $$\begin{array}{c} \frac{\partial G_{Bob}}{\partial P_{⋄B}}=\frac{\left(1-P_{2}\right)\left(3-7P_{1}\right)}{2} \end{array}$$

Here, we discuss it in three subcases.

(i) For $0<\frac{P_{2}\left(\mu +1\right)-2P_{1}P_{2}}{2P_{1}\left(1-P_{2}\right)}\leq 1$, we get $\frac{\mu +1}{\mu +5}\leq P_{1}\leq 1$. For μ≥2, it follows that $\frac{\mu +1}{\mu +5}\geq \frac{3}{7}$. It means that $\frac{\partial G_{Bob}}{\partial P_{⋄B}}<0$, i.e., $G_{Bob}$ decreases with $P_{⋄B}$. Thus, Bob will set $P_{⋄B}=0$ to maximize his gains. From Equation (A24), we get
  (A27) $$\begin{array}{c} G_{Bob_{1}}=2P_{1}-2P_{1}P_{2}-1 \end{array}$$
(ii) For $\frac{\left(\mu +1\right)P_{2}-2P_{1}P_{2}}{2P_{1}\left(1-P_{2}\right)}>1$, we can get $P_{1}<\frac{\mu +1}{\mu +5}$, and when $P_{1}\in \left[0,\frac{3}{7}\right]$, we get $\frac{\partial G_{Bob}}{\partial P_{⋄B}}\geq 0$, i.e., $G_{Bob}$ increases with $P_{⋄B}$. So, Bob makes $P_{⋄B}=1$ in order to maximize his gains. From Equation (A24), we can get that
  (A28) $$\begin{array}{c} G_{Bob_{2}}=-3P_{2}^{2}+3P_{2}-1 \end{array}$$
(iii) For $\frac{\left(\mu +1\right)P_{2}-2P_{1}P_{2}}{2P_{1}\left(1-P_{2}\right)}>1$, i.e., $P_{1}<\frac{\mu +1}{\mu +5}$, when $P_{1}\in \left(\frac{3}{7},\frac{\mu +1}{\mu +5}\right)$, we can get $\frac{\partial G_{Bob}}{\partial P_{⋄B}}<0$, i.e., $G_{Bob}$ decreases with $P_{⋄B}$. Thus, Bob will make $P_{⋄B}=0$ to maximize his gains. From Equation (A24), we obtain that
  (A29) $$\begin{array}{c} G_{Bob_{3}}=2P_{1}-2P_{1}P_{2}-1 \end{array}$$

**Case 2.**$\frac{\partial G_{Rice}}{\partial P_{⋄C}}\leq 0$, i.e., $\frac{\left(\mu +1\right)P_{2}-2P_{1}P_{2}}{2P_{1}\left(1-P_{2}\right)}\leq P_{⋄B}\leq 1$. For $0<\frac{P_{2}\left(\mu +1\right)-2P_{1}P_{2}}{2P_{1}\left(1-P_{2}\right)}\leq 1$, we get $\frac{\mu +1}{\mu +5}\leq P_{1}\leq 1$.

In this case, we have $P_{⋄C}=0$ and $P_{⋄B}=X_{0}$, where $X_{0}>\frac{\left(\mu +1\right)P_{2}-2P_{1}P_{2}}{2P_{1}\left(1-P_{2}\right)}$. From Equation (A24), we obtain that

(A30) $$\begin{array}{cc} G_{Bob_{4}} & =2P_{1}-1 \end{array}$$

If $\frac{\mu +1}{\mu +5}\leq P_{1}\leq 1$, we have $G_{Bob_{4}}>G_{Bob_{1}}$. Bob will make $P_{⋄B}=X_{0}$ to get the maximal gains for $\frac{\mu +1}{\mu +5}\leq P_{1}<1$, where $X_{0}>\frac{\left(\mu +1\right)P_{2}-2P_{1}P_{2}}{2P_{1}\left(1-P_{2}\right)}$.

**Table A1:** The values of $P_{⋄B}$ and $P_{⋄C}$. The values of $P_{B(C)}$ depend on the different cases in the game.

Cases	$0\leq P_{1}\leq \frac{3}{7}$	$\frac{3}{7}<P_{1}<\frac{\mu \;+\;1}{\mu \;+\;5}$	$\frac{\mu \;+\;1}{\mu \;+\;5}\leq P_{1}\leq 1$
$P_{⋄B}$	1	0	$X_{0}$
$P_{⋄C}$	1	1	0

## Appendix E. The Average Gain of Alice in the Second Classical Game

According to Table 5, we can get the average gain of Alice.

(A31) $$\begin{array}{cc} G_{Alice}= & -\left(\mu -1\right)P_{Rice_{1}}-P_{Bob_{1}}+2P_{Alice}-2P_{Rice_{2}}\\ = & -P_{⋄C}P_{2}\left(\mu -1\right)+P_{⋄B}P_{⋄C}\left(1-P_{2}\right)\left(2-3P_{2}\right)\\ \; & +2\left(1-P_{⋄C}\right)\left(1-2P_{1}\right)+2P_{⋄C}\left(1-P_{2}\right)(1\\ \; & -P_{⋄B})\left(1-2P_{1}\right) \end{array}$$

Here, three cases will be considered as follows.

**Case 1.** For $0\leq P_{1}\leq \frac{3}{7}$, we get $P_{⋄C}=1$ and $P_{⋄B}=1$. From Equation (A31), we get

(A32) $$\begin{array}{c} G_{Alice}=-P_{2}\left(\mu -1\right)+\left(1-P_{2}\right)\left(2-3P_{2}\right) \end{array}$$

which implies that

(A33) $$\begin{array}{c} \frac{dG_{Alice}}{dP_{1}}=-3P_{2}+\frac{\mu +4}{2} \end{array}$$

From μ≥2, we have $\frac{dG_{Alice}}{dP_{1}}\geq 0$, i.e., $G_{Alice}$ increases with $P_{1}$. It follows that $P_{1}=\frac{3}{7}$. We obtain that the maximum value $G_{Alice_{1}}$ of $G_{Alice}$ as

(A34) $$\begin{array}{c} G_{Alice_{1}}=\frac{54}{49}-\frac{2\mu }{7} \end{array}$$

**Case 2.** For $\frac{3}{7}<P_{1}<\frac{\mu +1}{\mu +5}$, we get $P_{⋄C}=1$ and $P_{⋄B}=0$. From Equation (A31), we get

(A35) $$\begin{array}{cc} G_{Alice} & =-\left(\mu -1\right)P_{2}+2\left(1-P_{2}\right)\left(1-2P_{1}\right)\\ \; & =-8P_{2}^{2}+\left(11-\mu \right)P_{2}-2 \end{array}$$

The partial derivative of $G_{Alice}$ with respect to $P_{2}=\frac{1-P_{1}}{2}$ is given by

(A36) $$\begin{array}{c} \frac{dG_{Alice}}{dP_{2}}=-16P_{2}+11-\mu \end{array}$$

From Equation (A36), $G_{Alice}$ increases with $P_{2}$ for $P_{2}\in \left[0,\frac{11-\mu }{16}\right)$, and decreases for $P_{2}\in \left(\frac{11-\mu }{16},1\right]$, thus the following three cases need to be considered according to it.

(i) For $\frac{11-\mu }{16}\leq \frac{2}{\mu +5}$, i.e., $\mu \geq 3+4\sqrt{2}$, $G_{Alice}$ decreases with $P_{2}$. So, $P_{2}=\frac{2}{\mu +5}$ and $P_{1}=\frac{\mu +1}{\mu +5}$. From Equation (A35), we get
  (A37) $$\begin{array}{c} G_{Alice_{2}}=-\frac{4\mu^{2}+8\mu +28}{{\left(\mu +5\right)}^{2}} \end{array}$$
(ii) For $\frac{11-\mu }{16}\geq \frac{2}{7}$, i.e., $2\leq \mu \leq \frac{45}{7}$, $G_{Alice}$ increases with $P_{2}$. So, $P_{2}=\frac{2}{7}$ and $P_{1}=\frac{3}{7}$. From Equation (A35), we get
  (A38) $$\begin{array}{cc} G_{Alice_{3}} & =\frac{24}{49}-\frac{2\mu }{7} \end{array}$$
(iii) For $\frac{2}{\mu +5}<\frac{11-\mu }{16}<\frac{2}{7}$, i.e., $\frac{45}{7}<\mu <3+4\sqrt{2}$, $G_{Alice}$ increases with $P_{2}$ for $P_{2}\in \left(\frac{2}{\mu +5},\frac{11-\mu }{16}\right)$, and decreases for $P_{2}\in \left(\frac{11-\mu }{16},\frac{2}{7}\right)$. So, $G_{Alice}$ takes its maximum at the point of $P_{2}=\frac{11-\mu }{16}$. From Equation (A35), we get
  (A39) $$\begin{array}{cc} G_{Alice_{4}} & =\frac{{\left(11-\mu \right)}^{2}}{32}-2 \end{array}$$

**Case 3.** For $\frac{\mu +1}{\mu +5}\leq P_{1}\leq 1$, we have $P_{⋄C}=0$ and $P_{⋄B}=X_{0}$, where $X_{0}>\frac{\left(\mu +1\right)P_{2}-2P_{1}P_{2}}{2P_{1}\left(1-P_{2}\right)}$. From Equation (A31), we get

(A40) $$\begin{array}{c} G_{Alice}=2-4P_{1} \end{array}$$

It is easy to prove that $G_{Alice}$ decreases with $P_{1}$. So, the maximum $G_{Alice_{5}}$ of $G_{Alice}$ achieves at $P_{1}=\frac{\mu +1}{\mu +5}$, which is given by

(A41) $$\begin{array}{c} G_{Alice_{5}}=\frac{6-2\mu }{\mu +5} \end{array}$$

It is easy to prove that $G_{Alice_{2}}<G_{Alice_{5}}$ for $\mu \geq 3+4\sqrt{2}$, and $G_{Alice_{3}}<G_{Alice_{1}}$ for $2\leq \mu <\frac{45}{7}$, and $G_{Alice_{4}}<G_{Alice_{1}}$ for $\frac{45}{7}<\mu <3+4\sqrt{2}$.

To sum up we get Table A2.

**Table A2:** The maximum $G_{Alice}^{m}$ of $G_{Alice}$ and the corresponding point $p_{\max}$.

Cases	$G_{{\mathrm{Alice}}_{1}}\geq G_{{\mathrm{Alice}}_{5}}$	$G_{{\mathrm{Alice}}_{1}}<G_{{\mathrm{Alice}}_{5}}$
$p_{\max}$	$\frac{3}{7}$	$\frac{\mu +1}{\mu +5}$
$G_{Alice}^{m}$	$G_{Alice_{1}}$	$G_{Alice_{5}}$

## Appendix F. Analysis of *β* and *γ* in the First Quantum Game

How Rice and Bob choose their own parameters to maximize their average gains? we will discuss them in five cases.

**Case 1.** For $x_{2}^{*}\leq 0$, Rice should take x=0 to maximize her gains. Since $x_{2}^{*}\geq x_{4}^{*}$, i.e., $x_{4}^{*}\leq 0$, Bob should take x=0 to maximize his gains. Hence, we have β=γ=0.

**Case 2.** For $x_{4}^{*}\geq 2$, Rice should take x=2 to maximize her gains. Since $x_{2}^{*}\geq x_{4}^{*}$, i.e., $x_{2}^{*}\geq 2$, Bob should take x=2 to maximize his gains. Therefore, we get β=γ=1.

**Case 3.** For $0<x_{2}^{*}\leq 1$, if Bob takes $\beta \geq x_{2}^{*}$, after knowing Bob’s actions, Rice will definitely choose γ=0 to maximize her gains, i.e., $x>x_{2}^{*}\geq x_{4}^{*}$. For Bob, when $x>x_{4}^{*}$, $G_{Bob}$ decreases with β. Hence, we have $\beta =x_{2}^{*}$ and γ=0. If Bob takes $\beta \leq x_{2}^{*}$, after knowing Bob’s actions, Rice will definitely choose $\gamma =x_{2}^{*}-\beta$ to maximize her gains. However, when $x=x_{2}^{*}$, $G_{Bob}$ decreases with β. In this case, we get β=0 and $\gamma =x_{2}^{*}$.

**Case 4.** For $x_{4}^{*}\leq 1<x_{2}^{*}$, if Bob takes $\beta <x_{2}^{*}-1$, after knowing Bob’s actions, Rice will definitely choose γ=1 to maximize her gains, i.e., $x_{4}^{*}\leq x<x_{2}^{*}$. The reason is that $G_{Bob}$ decreases with β for $x\geq x_{4}^{*}$. From Bob’s own gains, it should be β=0 and γ=1. If Bob takes $\beta \in [x_{2}^{*}-1,1]$, Rice will definitely choose $\gamma =x_{2}^{*}-\beta$ to maximize her gains after knowing Bob’s actions, i.e., $x=x_{2}^{*}\geq x_{4}^{*}$, because $G_{Bob}$ decreases with β for $x\geq x_{4}^{*}$. In this case, from Bob’s own interests, we have $\beta =x_{2}^{*}-1$ and γ=1. Note that $G_{Bob}$ is decreasing function in β for $x=x_{2}^{*}$. So, we get β=0 and γ=1.

**Case 5.** For $1<x_{4}^{*}<2$, if Bob takes $0\leq \beta \leq x_{4}^{*}-1$, Rice will choose γ=1 to maximize her gains after knowing Bob’s actions, i.e., $1\leq x\leq x_{4}^{*}$. It is because Bob gets the maximum value at $x=x_{4}^{*}$. We have $\beta =x_{4}^{*}-1$ and γ=1. If Bob takes $1\geq \beta >x_{4}^{*}-1$, Rice will choose γ as close as possible to $x_{2}^{*}$ to maximize her gains after knowing Bob’s actions, i.e., $x_{4}^{*}\leq x\leq x_{2}^{*}$. Thus, we obtain $\beta =x_{4}^{*}-1$ and γ=1.

## Appendix G. The Average Gain of Alice in the First Quantum Game

We can calculate Alice’s average gain according to the values of $x_{2}^{*}$ and $x_{4}^{*}$, and the first case of $x_{2}^{*}\leq 0$ has been discussed in Section 3.2.3, the remaining four cases are as follows.

**Case 1.** For $x_{4}^{*}\geq 2$, i.e., $\sqrt{\frac{2\alpha_{1}\omega_{1}}{3\alpha_{2}\omega_{2}}}-\frac{\omega_{1}}{\omega_{2}}\left(\sqrt{\frac{2}{3}}+1\right)\geq 2$, we get

(A42) $$\begin{array}{c} \frac{{\left(2+\left(\sqrt{\frac{2}{3}}+1\right)\frac{\omega_{1}}{\omega_{2}}\right)}^{2}}{{\left(2+\left(\sqrt{\frac{2}{3}}+1\right)\frac{\omega_{1}}{\omega_{2}}\right)}^{2}+\frac{4\omega_{1}}{3\omega_{2}}}\leq \alpha_{1}\leq 1 \end{array}$$

Let $E=\frac{{\left(2+\left(\sqrt{\frac{2}{3}}+1\right)\frac{\omega_{1}}{\omega_{2}}\right)}^{2}}{{\left(2+\left(\sqrt{\frac{2}{3}}1\right)\frac{\omega_{1}}{\omega_{2}}\right)}^{2}+\frac{4\omega_{1}}{3\omega_{2}}}$, i.e., $E\leq \alpha_{1}\leq 1$. In this case, we have β=γ=1. From Equation (36) we get

(A43) $$\begin{array}{c} G_{Alice}=\left(\eta +3\right){\left(\sqrt{\alpha_{1}\omega_{1}}+2\sqrt{\alpha_{2}\omega_{2}}\right)}^{2}-\eta -1 \end{array}$$

The derivative of $G_{Alice}$ with respect to $\alpha_{1}$ is given by

(A44) $$\begin{array}{c} \frac{dG_{Alice}}{d\alpha_{1}}=\left(\eta +3\right)\left(\frac{\sqrt{2\omega_{1}\omega_{2}}\left(1-2\alpha_{1}\right)}{\sqrt{\alpha_{1}-\alpha_{1}^{2}}}+\omega_{1}-2\omega_{2}\right) \end{array}$$

The second derivative of $G_{Alice}$ with respect to $\alpha_{1}$ is given by

(A45) $$\begin{array}{c} \frac{d^{2}G_{Alice}}{d\alpha_{1}^{2}}=-\frac{\sqrt{\omega_{1}\omega_{2}}\left(\eta +3\right)}{\sqrt{2}{\left(\alpha_{1}-\alpha_{1}^{2}\right)}^{\frac{3}{2}}} \end{array}$$

From Equation (A45), we know that $\frac{d^{2}G_{Alice}}{d\alpha_{1}^{2}}<0$, and $\frac{dG_{Alice}}{d\alpha_{1}}$ has at most one zero when $0\leq \alpha_{1}\leq 1$. From $\frac{dG_{Alice}}{d\alpha_{1}}=0$, it follows that

(A46) $$\begin{array}{c} \frac{1-2\alpha_{1}}{\sqrt{\alpha_{1}-\alpha_{1}^{2}}}=\frac{\left(2\omega_{2}-\omega_{1}\right)}{\sqrt{2\omega_{1}\omega_{2}}}. \end{array}$$

Denote $F=\frac{\left(2\omega_{2}-\omega_{1}\right)}{\sqrt{2\omega_{1}\omega_{2}}}$. If F≥0, i.e., $\omega_{1}\leq 2\omega_{2}$, the solution of Equation (A46) is given by $\alpha =0.5-\frac{F}{2\sqrt{F^{2}+4}}=\omega_{1}$. $G_{Alice}$ is increasing function for $\alpha_{1}\in \left(0,\omega_{1}\right)$, while it is decreasing for $\alpha_{1}\in \left(\omega_{1},1\right)$. From $\alpha_{1}\geq E>\omega_{1}$, $G_{Alice}$ achieves the maximum value at $\alpha_{1}=E$. If F<0, i.e., $\omega_{1}>2\omega_{2}$, the solution of Equation (A46) is given by $\alpha =0.5-\frac{F}{2\sqrt{F^{2}+4}}=2\omega_{2}$. $G_{Alice}$ is increasing function for $\alpha_{1}\in \left(0,2\omega_{2}\right)$, and decreasing for $\alpha_{1}\in \left(2\omega_{2},1\right)$. From $\alpha_{1}\geq E>\omega_{1}>2\omega_{2}$, $G_{Alice}$ gets the maximum value at $\alpha_{1}=E$. It means that $G_{Alice}$ gets the maximum value $G_{Alice_{2}}$ as

(A47) $$\begin{array}{c} G_{Alice_{2}}=\left(\eta +3\right){\left(\sqrt{\omega_{1}E}+\sqrt{2\omega_{2}\left(1-E\right)}\right)}^{2}-\eta -1 \end{array}$$

For $x_{4}^{*}\geq 2$, where the corresponding point is denoted by $p_{2}$.

**Case 2.** For $0<x_{2}^{*}\leq 1$, i.e., $0<\sqrt{\frac{\alpha_{1}\omega_{1}\left(\eta +1\right)}{3\alpha_{2}\omega_{2}}}-\left(\sqrt{\frac{\eta +1}{3}}+1\right)\frac{\omega_{1}}{\omega_{2}}\leq 1$, we get

(A48) $$\begin{array}{c} D<\alpha_{1}\leq G \end{array}$$

where $G=\frac{{\left(1+\left(\sqrt{\frac{\eta +1}{3}}+1\right)\frac{\omega_{1}}{\omega_{2}}\right)}^{2}}{{\left(1+\left(\sqrt{\frac{\eta +1}{3}}+1\right)\frac{\omega_{1}}{\omega_{2}}\right)}^{2}+\frac{2\omega_{1}\left(\eta +1\right)}{3\omega_{2}}}$. In this case, we have β=0 and $\gamma =x_{2}^{*}$. From Equation (36) we have

(A49) $$\begin{array}{cc} G_{Alice}= & \frac{\left(\eta +3\right){\left(\sqrt{\alpha_{1}\omega_{1}}+x_{2}^{*}\sqrt{\alpha_{2}\omega_{2}}\right)}^{2}}{\omega_{1}+\omega_{2}x_{2}^{*}}-x_{2}^{*}\eta \alpha_{2}-1-\eta \alpha_{1}\\ = & \frac{\sqrt{\alpha_{2}}\left(\eta +3\right){\left(1+\sqrt{\frac{\eta +1}{3}}\right)}^{2}{\left(\sqrt{\frac{\alpha_{1}\omega_{1}}{\omega_{2}}}-\frac{\omega_{1}\sqrt{\alpha_{2}}}{\omega_{2}}\right)}^{2}}{\sqrt{\frac{\eta +1}{3}}\left(\sqrt{\frac{\alpha_{1}\omega_{1}}{\omega_{2}}}-\frac{\omega_{1}\sqrt{\alpha_{2}}}{\omega_{2}}\right)}\\ \; & -\eta \sqrt{\frac{\left(\eta +1\right)\alpha_{1}\alpha_{2}\omega_{1}}{3\omega_{2}}}+\frac{\eta \omega_{1}\alpha_{2}\left(\sqrt{\frac{\eta +1}{3}}+1\right)}{\omega_{2}}\\ \; & -\eta \alpha_{1}-1 \end{array}$$

From $D<\alpha_{1}<G$, we have $\sqrt{\frac{\alpha_{1}\omega_{1}}{\omega_{2}}}-\frac{\omega_{1}\sqrt{\alpha_{2}}}{\omega_{2}}\neq 0$. Equation (A49) is rewritten into

(A50) $$\begin{array}{cc} G_{Alice}= & \left(\frac{2\sqrt{3}\eta +4\sqrt{3}}{\sqrt{\eta +1}}+2\eta +6\right)\left(\sqrt{\frac{\omega_{1}\alpha_{1}\alpha_{2}}{\omega_{2}}}-\frac{\omega_{1}\alpha_{2}}{\omega_{2}}\right)\\ \; & -\eta \alpha_{1}-1+\frac{\eta \omega_{1}\alpha_{2}}{\omega_{2}} \end{array}$$

The derivative of $G_{Alice}$ with respect to $\alpha_{1}$ is given by

(A51) $$\begin{array}{cc} \frac{dG_{Alice}}{d\alpha_{1}}= & \left(\frac{2\sqrt{3}\eta +4\sqrt{3}}{\sqrt{\eta +1}}+2\eta +6\right)\\ \; & \times \left(\frac{\sqrt{\omega_{1}}\left(1-2\alpha_{1}\right)}{\sqrt{8\omega_{2}\left(\alpha_{1}-\alpha_{2}^{2}\right)}}+\frac{\omega_{1}}{2\omega_{2}}\right)-\frac{\eta \omega_{1}}{2\omega_{2}}-\eta \end{array}$$

The second derivative of $G_{Alice}$ with respect to $\alpha_{1}$ is given by

(A52) $$\begin{array}{cc} \frac{d^{2}G_{Alice}}{d\alpha_{1}^{2}}= & -(\frac{2\sqrt{3}\eta +4\sqrt{3}}{\sqrt{\eta +1}}+2\eta +6)\\ \; & \times \sqrt{\frac{\omega_{1}}{32\omega_{2}{\left(\alpha_{1}-\alpha_{1}^{2}\right)}^{3}}} \end{array}$$

From Equation (A52), we obtain that $\frac{d^{2}G_{Alice}}{d\alpha_{1}^{2}}<0$, and $\frac{dG_{Alice}}{d\alpha_{1}}=0$ has at most one solution when $0\leq \alpha_{1}\leq 1$. By letting $\frac{dG_{Alice}}{d\alpha_{1}}=0$, we get

(A53) $$\begin{array}{c} \frac{1-2\alpha_{1}}{\sqrt{\alpha_{1}-\alpha_{1}^{2}}}=\frac{2\sqrt{2}\eta \omega_{2}-\sqrt{2}\omega_{1}\left(\frac{2\sqrt{3}\eta +4\sqrt{3}}{\sqrt{\eta +1}}+\eta +6\right)}{\left(\frac{2\sqrt{3}\eta +4\sqrt{3}}{\sqrt{\eta +1}}+2\eta +6\right)\sqrt{\omega_{1}\omega_{2}}} \end{array}$$

Denote

(A54) $$\begin{array}{c} H=\frac{2\sqrt{2}\eta \omega_{2}-\sqrt{2}\omega_{1}\left(\frac{2\sqrt{3}\eta +4\sqrt{3}}{\sqrt{\eta +1}}+\eta +6\right)}{\left(\frac{2\sqrt{3}\eta +4\sqrt{3}}{\sqrt{\eta +1}}+2\eta +6\right)\sqrt{\omega_{1}\omega_{2}}} \end{array}$$

The solution of Equation (A53) is given by $\alpha =0.5-\frac{H}{2\sqrt{H^{2}+4}}$. $G_{Alice}$ is increasing function when $\alpha_{1}\in \left(0,\alpha \right)$, or decreasing for $\alpha_{1}\in \left(\alpha ,1\right)$. Thus, $G_{Alice}$ gets the maximum value at $\alpha_{1}=D$ if α≤D, or gets the maximum value at $\alpha_{1}=G$ if α≥G. Moreover, $G_{Alice}$ gets the maximum value at $\alpha_{1}=\alpha$ if D<α<G. The maximum gain of Alice is denoted by $G_{Alice_{3}}$ when $0<x_{2}^{*}\leq 1$, where the corresponding point is denoted by $p_{3}$.

**Case 3.** For $x_{4}^{*}\leq 1<x_{2}^{*}$, i.e., $\sqrt{\frac{\left(\eta +1\right)\alpha_{1}\omega_{1}}{3\alpha_{2}\omega_{2}}}-\left(\sqrt{\frac{\eta +1}{3}}+1\right)\frac{\omega_{1}}{\omega_{2}}>1$ and $\sqrt{\frac{2\alpha_{1}\omega_{1}}{3\alpha_{2}\omega_{2}}}-\left(\sqrt{\frac{2}{3}}+1\right)\frac{\omega_{1}}{\omega_{2}}\leq 1$, we get

(A55) $$\begin{array}{c} G<\alpha_{1}\leq K \end{array}$$

where $K=\frac{{\left(1+\left(\sqrt{\frac{2}{3}}+1\right)\frac{\omega_{1}}{\omega_{2}}\right)}^{2}}{{\left(1+\left(\sqrt{\frac{2}{3}}+1\right)\frac{\omega_{1}}{\omega_{2}}\right)}^{2}+\frac{4\omega_{1}}{3\omega_{2}}}$. In this case, we have β=0 and γ=1. From Equation (36) we get

(A56) $$\begin{array}{c} G_{Alice}=\frac{\left(\eta +3\right){\left(\sqrt{\alpha_{1}\omega_{1}}+\sqrt{\alpha_{2}\omega_{2}}\right)}^{2}}{\omega_{1}+\omega_{2}}-\eta \alpha_{2}-\eta \alpha_{1}-1 \end{array}$$

The derivative of $G_{Alice}$ with respect to $\alpha_{1}$ is given by

(A57) $$\begin{array}{c} \frac{dG_{Alice}}{d\alpha_{1}}=\frac{\left(\eta +3\right)\left(\omega_{1}-\frac{\omega_{2}}{2}+\frac{\sqrt{\omega_{1}\omega_{2}}\left(1-2\alpha_{1}\right)}{\sqrt{2(\alpha_{1}-\alpha_{1}^{2})}}\right)}{\omega_{1}+\omega_{2}}-\frac{\eta }{2} \end{array}$$

The second derivative of $G_{Alice}$ with respect to $\alpha_{1}$ is given by

(A58) $$\begin{array}{c} \frac{d^{2}G_{Alice}}{d\alpha_{1}^{2}}=-\frac{\left(\eta +3\right)\sqrt{\omega_{1}\omega_{2}}}{\sqrt{2}\left(\omega_{1}+\omega_{2}\right){\left(\alpha_{1}-\alpha_{1}^{2}\right)}^{\frac{3}{2}}} \end{array}$$

From Equation (A58), we know that $\frac{d^{2}G_{Alice}}{d\alpha_{1}^{2}}<0$, and $\frac{dG_{Alice}}{d\alpha_{1}}$ has at most one zero when $0\leq \alpha_{1}\leq 1$. By letting $\frac{dG_{Alice}}{d\alpha_{1}}=0$, we get

(A59) $$\begin{array}{c} \frac{1-2\alpha_{1}}{\sqrt{\alpha_{1}-\alpha_{1}^{2}}}=\frac{\left(2\eta +3\right)\omega_{2}-\left(\eta +6\right)\omega_{1}}{\sqrt{2\omega_{1}\omega_{2}}\left(\eta +3\right)} \end{array}$$

Let $M=\frac{\left(2\eta +3\right)\omega_{2}-\left(\eta +6\right)\omega_{1}}{\sqrt{2\omega_{1}\omega_{2}}\left(\eta +3\right)}$. If M≥0, the solution of Equation (A59) is given by $\alpha =0.5-\frac{M}{2\sqrt{M^{2}+4}}$. $G_{Alice}$ is increasing function for $\alpha_{1}\in \left(0,\alpha \right)$, while it is decreasing for $\alpha_{1}\in \left(\alpha ,1\right)$. Thus, $G_{Alice}$ gets the maximum value at $\alpha_{1}=G$ if α≤G, or at $\alpha_{1}=K$ if α≥K. Moreover, $G_{Alice}$ gets the maximum value at $\alpha_{1}=\alpha$ if G<α<K. The maximum gain of Alice gets is denoted by $G_{Alice_{4}}$ when $x_{2}^{*}>1\geq x_{4}^{*}$, where the corresponding point is denoted by $p_{4}$.

**Case 4.** For $1<x_{4}^{*}<2$, i.e., $1<\sqrt{\frac{2\alpha_{1}\omega_{1}}{3\alpha_{2}\omega_{2}}}-\left(\sqrt{\frac{2}{3}}+1\right)\frac{\omega_{1}}{\omega_{2}}<2$, we get

(A60) $$\begin{array}{c} K<\alpha_{1}<E \end{array}$$

where $E=\frac{{\left(2+\left(\sqrt{\frac{2}{3}}+1\right)\frac{\omega_{1}}{\omega_{2}}\right)}^{2}}{{\left(2+\left(\sqrt{\frac{2}{3}}+1\right)\frac{\omega_{1}}{\omega_{2}}\right)}^{2}+\frac{4\omega_{1}}{3\omega_{2}}}$. In this case, we have $\beta =x_{4}^{*}-1$ and γ=1. From Equation (36) we get

(A61) $$\begin{array}{cc} G_{Alice}= & \frac{\left(\eta +3\right){\left(\sqrt{\alpha_{1}\omega_{1}}+x_{4}^{*}\sqrt{\alpha_{2}\omega_{2}}\right)}^{2}}{\omega_{1}+\omega_{2}x_{4}^{*}}-x_{4}^{*}\eta \alpha_{2}-1-\eta \alpha_{1}\\ = & \left(\frac{\sqrt{3}\eta +5\sqrt{3}}{\sqrt{2}}+2\eta +6\right)\left(\sqrt{\frac{\omega_{1}\alpha_{1}\alpha_{2}}{\omega_{2}}}-\frac{\omega_{1}\alpha_{2}}{\omega_{2}}\right)\\ \; & -\eta \alpha_{1}-1+\frac{\eta \omega_{1}\alpha_{2}}{\omega_{2}} \end{array}$$

The derivative of $G_{Alice}$ with respect to $\alpha_{1}$ is given by

(A62) $$\begin{array}{cc} \frac{dG_{Alice}}{d\alpha_{1}}= & \left(\frac{\sqrt{3}\eta +5\sqrt{3}}{\sqrt{2}}+2\eta +6\right)\\ \; & \times \left(\frac{\sqrt{\omega_{1}}\left(1-2\alpha_{1}\right)}{\sqrt{8\omega_{2}\left(\alpha_{1}-\alpha_{2}^{2}\right)}}+\frac{\omega_{1}}{2\omega_{2}}\right)-\frac{\eta \omega_{1}}{2\omega_{2}}-\eta \end{array}$$

The second derivative of $G_{Alice}$ with respect to $\alpha_{1}$ is given by

(A63) $$\begin{array}{c} \frac{d^{2}G_{Alice}}{d\alpha_{1}^{2}}=-\left(\frac{\sqrt{3}\left(\eta +5\right)}{\sqrt{2}}+2\eta +6\right)\frac{\sqrt{\omega_{1}}}{\sqrt{32\omega_{2}}{\left(\alpha_{1}-\alpha_{1}^{2}\right)}^{\frac{3}{2}}} \end{array}$$

From Equation (A63), we have that $\frac{d^{2}G_{Alice}}{d\alpha_{1}^{2}}<0$, and $\frac{dG_{Alice}}{d\alpha_{1}}$ has at most one zero when $0\leq \alpha_{1}\leq 1$. By setting $\frac{dG_{Alice}}{d\alpha_{1}}=0$, we get

(A64) $$\begin{array}{c} \frac{1-2\alpha_{1}}{\sqrt{\alpha_{1}-\alpha_{1}^{2}}}=\frac{4\eta \omega_{2}-\sqrt{6}\omega_{1}\left(\eta +5\right)-2\omega_{1}\left(\eta +6\right)}{\left(\eta +5\right)\sqrt{3\omega_{1}\omega_{2}}+\left(2\eta +6\right)\sqrt{2\omega_{1}\omega_{2}}} \end{array}$$

Let $N=\frac{4\eta \omega_{2}-\sqrt{6}\omega_{1}\left(\eta +5\right)-2\omega_{1}\left(\eta +6\right)}{\left(\eta +5\right)\sqrt{3\omega_{1}\omega_{2}}+\left(2\eta +6\right)\sqrt{2\omega_{1}\omega_{2}}}$. The solution of Equation (A64) is given by $\alpha =0.5-\frac{N}{2\sqrt{N^{2}+4}}$. $G_{Alice}$ is increasing for $\alpha_{1}\in \left(0,\alpha \right)$, and decreasing for $\alpha_{1}\in \left(\alpha ,1\right)$. $G_{Alice}$ is increasing for $\alpha_{1}\in \left(0,\alpha \right)$, and decreasing for $\alpha_{1}\in \left(\alpha ,1\right)$. Thus, $G_{Alice}$ gets the maximum value at $\alpha_{1}=K$ if α≤K, or at $\alpha_{1}=E$ if α≥E. Moreover, $G_{Alice}$ gets the maximum value at $\alpha_{1}=\alpha$ if K<α<E. The maximum gain of Alice is denoted by $G_{Alice_{5}}$ when $1<x_{4}^{*}<2$, where the corresponding point is denoted by $p_{5}$.

## Appendix H. The Average Gain of Rice in the Second Quantum Game

According to Table A2, we get

(A65) $$\begin{array}{cc} G_{Rice}= & \mu P_{Rice_{1}}+P_{Rice_{2}}-P_{Bob_{1}}-P_{Alice}\\ = & -2\frac{{\left(\sqrt{\alpha_{1}\omega_{1}}+x\sqrt{\alpha_{2}\omega_{2}}\right)}^{2}}{\omega_{1}+\omega_{2}x}+\left(\mu -1\right)\alpha_{2}\left(1-\gamma \right)\\ \; & +\alpha_{1}+2\alpha_{2}\beta \end{array}$$

where x=β+γ. The partial derivative of $G_{Rice}$ with respect to γ is given by

(A66) $$\begin{array}{cc} \frac{\partial G_{Rice}}{\partial \gamma }= & \frac{-\left(\mu +1\right)\omega_{2}^{2}\alpha_{2}x^{2}-2\left(\mu +1\right)\omega_{1}\omega_{2}\alpha_{2}x}{{\left(\omega_{1}+\omega_{2}x\right)}^{2}}\\ \; & +\frac{\left(1-\mu \right)\omega_{1}^{2}\alpha_{2}-4\omega_{1}\sqrt{\alpha_{1}\alpha_{2}\omega_{1}\omega_{2}}+2\omega_{1}\omega_{2}\alpha_{1}}{{\left(\omega_{1}+\omega_{2}x\right)}^{2}} \end{array}$$

By setting $\frac{\partial G_{Rice}}{\partial \gamma }=0$, we get its solutions as

(A67) $$\begin{array}{cc} x_{1}^{*} & =-\sqrt{\frac{2\omega_{1}}{\left(\mu +1\right)\alpha_{2}}}\frac{\left(\sqrt{\alpha_{1}\omega_{2}}-\sqrt{\alpha_{2}\omega_{1}}\right)}{\omega_{2}}-\frac{\omega_{1}}{\omega_{2}}\\ x_{2}^{*} & =\sqrt{\frac{2\omega_{1}}{\left(\mu +1\right)\alpha_{2}}}\frac{\left(\sqrt{\alpha_{1}\omega_{2}}-\sqrt{\alpha_{2}\omega_{1}}\right)}{\omega_{2}}-\frac{\omega_{1}}{\omega_{2}} \end{array}$$

From Equation (A67), we know that $x_{1}^{*}\leq 0$ and $x_{2}^{*}\geq x_{1}^{*}$. From Equations (A66) and (A67), $G_{Rice}$ is decreasing for $x\leq x_{1}^{*}$ or $x>x_{2}^{*}$, and increasing for $x_{1}^{*}<x\leq x_{2}^{*}$. When, $G_{Rice}$ is decreasing in *x*. Since 0≤x≤2, when $x_{2}^{*}\leq 0$, $G_{Rice}$ achieves its maximum at x=0. When $x_{2}^{*}\geq 2$, $G_{Rice}$ gets its maximum at x=2. When $0<x_{2}^{*}<2$, $G_{Rice}$ gets the maximum at $x=x_{2}^{*}$.

## Appendix I. The Average Gain of Bob in the Second Quantum Game

According to Table A2, we get that

(A68) $$\begin{array}{cc} G_{Bob}= & 2P_{Bob_{1}}+P_{Bob_{2}}-P_{Rice_{1}}-P_{Alice}\\ = & -2\frac{{\left(\sqrt{\alpha_{1}\omega_{1}}+x\sqrt{\alpha_{2}\omega_{2}}\right)}^{2}}{\left(\omega_{1}+\omega_{2}x\right)}\\ \; & +\alpha_{1}+\alpha_{2}-\alpha_{2}\beta +2\alpha_{2}\gamma \end{array}$$

The partial derivative of $G_{Bob}$ with respect to β is given by

(A69) $$\begin{array}{cc} \frac{\partial G_{Bob}}{\partial \beta }= & \frac{-3\omega_{2}^{2}\alpha_{2}x^{2}-6\omega_{1}\omega_{2}\alpha_{2}x-\omega_{1}^{2}\alpha_{2}}{{\left(\omega_{1}+\omega_{2}x\right)}^{2}}\\ \; & +\frac{-4\omega_{1}\sqrt{\alpha_{1}\alpha_{2}\omega_{1}\omega_{2}}+2\omega_{1}\omega_{2}\alpha_{1}}{{\left(\omega_{1}+\omega_{2}x\right)}^{2}} \end{array}$$

By setting $\frac{\partial G_{Bob}}{\partial \beta }=0$, we get two solutions as

(A70) $$\begin{array}{cc} x_{3}^{*} & =-\sqrt{\frac{2\omega_{1}}{3\alpha_{2}}}\frac{|\sqrt{\alpha_{1}\omega_{2}}-\sqrt{\alpha_{2}\omega_{1}}|}{\omega_{2}}-\frac{\omega_{1}}{\omega_{2}}\\ x_{4}^{*} & =\sqrt{\frac{2\omega_{1}}{3\alpha_{2}}}\frac{|\sqrt{\alpha_{1}\omega_{2}}-\sqrt{\alpha_{2}\omega_{1}}|}{\omega_{2}}-\frac{\omega_{1}}{\omega_{2}} \end{array}$$

It follows that $\alpha_{1}\geq \omega_{1}$. Equation (A70) is then written into

(A71) $$\begin{array}{cc} x_{3}^{*} & =-\sqrt{\frac{2\omega_{1}}{3\alpha_{2}}}\frac{\sqrt{\alpha_{1}\omega_{2}}-\sqrt{\alpha_{2}\omega_{1}}}{\omega_{2}}-\frac{\omega_{1}}{\omega_{2}}\\ x_{4}^{*} & =\sqrt{\frac{2\omega_{1}}{3\alpha_{2}}}\frac{\sqrt{\alpha_{1}\omega_{2}}-\sqrt{\alpha_{2}\omega_{1}}}{\omega_{2}}-\frac{\omega_{1}}{\omega_{2}} \end{array}$$

From Equation (A71), we obtain $x_{3}^{*}\leq 0$ and $x_{4}^{*}\geq x_{3}^{*}$. From Equations (A69) and (A71), $G_{Bob}$ decreases with $x\leq x_{3}^{*}$ or $x>x_{4}^{*}$, and increases for $x_{3}^{*}<x\leq x_{4}^{*}$. Since 0≤x≤2, $G_{Bob}$ achieves its maximum at x=0 when $x_{4}^{*}\leq 0$. $G_{Bob}$ gets its maximum at x=2 when $x_{4}^{*}\geq 2$. When $0<x_{4}^{*}<2$, $G_{Bob}$ gets the maximum at $x=x_{4}^{*}$. From P≥2, we obtain $x_{4}^{*}\geq x_{2}^{*}$.

Now, we make a brief analysis of the strategy of Rice and Bob as follows.

**Case 1.** For $x_{4}^{*}\leq 0$, both Rice and Bob should take x=0 to maximize its own gain from $x_{4}^{*}\geq x_{2}^{*}$, i.e., $x_{2}^{*}\leq 0$. It follows that β=γ=0.

**Case 2.** For $x_{2}^{*}\geq 2$, both Rice and Bob should take x=2 to maximize the gain from $x_{4}^{*}\geq x_{2}^{*}$, i.e., $x_{4}^{*}\geq 2$. Therefore, we have β=γ=1.

**Case 3.** For $x_{2}^{*}\leq 0<x_{4}^{*}\leq 1$, we have $x\geq 0>x_{2}^{*}$ for any β being chosen by Bob. Rice will choose γ=0 to maximize her gain. For Bob, $G_{Bob}$ increases with β when $x<x_{4}^{*}$, and decreases with β when $x>x_{4}^{*}$, So, we have $\beta =x_{4}^{*}$ and γ=0.

**Case 4.** For $0<x_{2}^{*}\leq x_{4}^{*}\leq 1$ and $x_{2}^{*}<\beta \leq 1$, we have $x>x_{2}^{*}$ for any β being chosen by Bob. Rice will choose γ=0 to maximize her gain, i.e., $\beta =x_{4}^{*}$. From Equation (A68), we get that

(A72) $$\begin{array}{c} G_{Bob_{1}}=\alpha_{1}+\alpha_{2}-\alpha_{2}x_{4}^{*}-2\frac{{\left(\sqrt{\alpha_{1}\omega_{1}}+x_{4}^{*}\sqrt{\alpha_{2}\omega_{2}}\right)}^{2}}{\omega_{1}+\omega_{2}x_{4}^{*}} \end{array}$$

**Case 5.** For $0<x_{2}^{*}\leq x_{4}^{*}\leq 1$ and $0\leq \beta \leq x_{2}^{*}$, we have $x\leq x_{2}^{*}$ for any β being chosen by Bob. Rice will choose $\gamma =x_{2}^{*}-\beta$ to maximize her gain from $x=x_{2}^{*}$. $G_{Bob}$ decreases with β. It implies β=0 and $\gamma =x_{2}^{*}$. From Equation (A68), we get that

(A73) $$\begin{array}{c} G_{Bob_{2}}=\alpha_{1}+\alpha_{2}+2\alpha_{2}x_{2}^{*}-2\frac{{\left(\sqrt{\alpha_{1}\omega_{1}}+x_{2}^{*}\sqrt{\alpha_{2}\omega_{2}}\right)}^{2}}{\omega_{1}+\omega_{2}x_{2}^{*}} \end{array}$$

Thus if $G_{Bob_{1}}\geq G_{Bob_{2}}$, we get that $\beta =x_{4}^{*}$ and γ=0. Otherwise, β=0 and $\gamma =x_{2}^{*}$.

**Case 6.** For $x_{2}^{*}\leq 0$ and $1<x_{4}^{*}$, we get that $x>0>x_{2}^{*}$ for any β being chosen by Bob. Rice will choose γ=0 to maximize her gain. For Bob, $G_{Bob}$ increases with β when $x<x_{4}^{*}$. So, β=1 and γ=0.

**Case 7.** For $0<x_{2}^{*}<1<x_{4}^{*}$ and $x_{2}^{*}<\beta \leq 1$, we have $x>x_{2}^{*}$ for any β being chosen by Bob. Rice will choose γ=0 to maximize her gain. We get that β=1 and γ=0. From Equation (A68), it implies that

(A74) $$\begin{array}{c} G_{Bob_{3}}=\alpha_{1}-2\frac{{\left(\sqrt{\alpha_{1}\omega_{1}}+\sqrt{\alpha_{2}\omega_{2}}\right)}^{2}}{\omega_{1}+\omega_{2}} \end{array}$$

**Case 8.** For $0<x_{2}^{*}<1<x_{4}^{*}$ and $0\leq \beta \leq x_{2}^{*}$, we obtain that $x\leq x_{2}^{*}$ for any β being chosen by Bob. Rice will choose $\gamma =x_{2}^{*}-\beta$ to maximize her gain because of $x=x_{2}^{*}$. $G_{Bob}$ decreases with β. In this case, we get that β=0 and $\gamma =x_{2}^{*}$. From Equation (A68), we obtain that

(A75) $$\begin{array}{c} G_{Bob_{4}}=\alpha_{1}+\alpha_{2}+2\alpha_{2}x_{2}^{*}-2\frac{{\left(\sqrt{\alpha_{1}\omega_{1}}+x_{2}^{*}\sqrt{\alpha_{2}\omega_{2}}\right)}^{2}}{\omega_{1}+\omega_{2}x_{2}^{*}} \end{array}$$

Hence, if $G_{Bob_{3}}\geq G_{Bob_{4}}$, we obtain that β=1 and γ=0. Otherwise, β=0 and $\gamma =x_{2}^{*}$.

**Case 9.** For $1\leq x_{2}^{*}<2$, Rice will choose γ=1 to maximize her gain if Bob chooses $\beta \leq x_{2}^{*}-1$, i.e., $x<x_{2}^{*}$. So, $\beta =x_{2}^{*}-1$ and γ=1. If Bob chooses $\beta \geq x_{2}^{*}-1$, Rice will choose $\gamma =x_{2}^{*}-\beta$ to maximize her gain. Note that $G_{Bob}$ decreases with β when $x=x_{2}^{*}$. It follows that $\beta =x_{2}^{*}-1$ and γ=1. Thus, $\beta =x_{2}^{*}-1$ and γ=1.

To sum up, we obtain that

$$\begin{array}{c} \left(\beta ,\gamma \right)=\left\{\begin{array}{ccc} (0,0), & \mathrm{if} & x_{4}^{*}\leq 0;\\ (x_{4}^{*},0), & \mathrm{if} & x_{2}^{*}\leq 0<x_{4}^{*}\leq 1;\\ (x_{4}^{*},0), & \mathrm{if} & 0<x_{2}^{*}\leq x_{4}^{*}\leq 1,\\ & & G_{Bob_{1}}\geq G_{Bob_{2}};\\ (0,x_{2}^{*}), & \mathrm{if} & 0<x_{2}^{*}\leq x_{4}^{*}\leq 1,\\ & & G_{Bob_{1}}<G_{Bob_{2}};\\ (1,0), & \mathrm{if} & 1<x_{4}^{*},x_{2}^{*}\leq 0;\\ (1,0), & \mathrm{if} & 0<x_{2}^{*}<1<x_{4}^{*},\\ & & G_{Bob_{3}}\geq G_{Bob_{4}};\\ (0,x_{2}^{*}), & \mathrm{if} & 0<x_{2}^{*}<1<x_{4}^{*},\\ & & G_{Bob_{3}}<G_{Bob_{4}};\\ (x_{2}^{*}-1,1), & \mathrm{if} & 1\leq x_{2}^{*}<2;\\ \left(1,1\right) & \mathrm{if} & x_{2}^{*}\geq 2. \end{array}\right. \end{array}$$

## Appendix J. The Average Gain of Alice in the Second Quantum Game

According to Table A2, we have

(A76) $$\begin{array}{cc} G_{Alice}= & 2P_{Alice}-2P_{Rice_{2}}-P_{Bob_{1}}-\left(\mu -1\right)P_{Rice_{1}}\\ = & \frac{4{\left(\sqrt{\alpha_{1}\omega_{1}}+x\sqrt{\alpha_{2}\omega_{2}}\right)}^{2}}{\omega_{1}+\omega_{2}x}-\mu \alpha_{2}\\ \; & +\left(\mu -3\right)\alpha_{2}\gamma -2\alpha_{1}-\alpha_{2}\beta \end{array}$$

where x=β+γ.

The value of $G_{Alice}$ varies with the different values of $x_{2}^{*}$ and $x_{4}^{*}$, we can calculate Alice’s average gain in the following nine cases according to that.

**Case 1.** For $x_{4}^{*}\leq 0$, it follows that $\sqrt{\frac{2\alpha_{1}\omega_{1}}{3\alpha_{2}\omega_{2}}}-\left(\sqrt{\frac{2}{3}}+1\right)\frac{\omega_{1}}{\omega_{2}}\leq 0$. We get

(A77) $$\begin{array}{c} \omega_{1}\leq \alpha_{1}\leq D \end{array}$$

where $D=\frac{{\left(\sqrt{\frac{2}{3}}+1\right)}^{2}}{{\left(\sqrt{\frac{2}{3}}+1\right)}^{2}+\frac{4\omega_{2}}{3\omega_{1}}}$. In this case, β=γ=0. From Equation (A76), we obtain

(A78) $$\begin{array}{c} G_{Alice}=2\alpha_{1}-\mu \alpha_{2} \end{array}$$

From $\frac{\partial G_{Alice}}{\partial \alpha_{1}}=2+\frac{\mu }{2}$, we get that $G_{Alice}$ increases with $\alpha_{1}$ when $\omega_{1}\leq \alpha_{1}\leq D$. So, $G_{Alice}$ gets the maximum at $\alpha_{1}=D$, which is denoted by $G_{Alice_{1}}$ given by

(A79) $$\begin{array}{c} G_{Alice_{1}}=\frac{D(4+\mu )-\mu }{2} \end{array}$$

where the corresponding point is denoted by $p_{1}$.

**Case 2.** For $x_{2}^{*}\geq 2$, it follows that $\sqrt{\frac{2\alpha_{1}\omega_{1}}{\alpha_{2}\omega_{2}\left(\mu +1\right)}}-\left(\sqrt{\frac{2}{\left(\mu +1\right)}}+1\right)\frac{\omega_{1}}{\omega_{2}}\geq 2$. We get

(A80) $$\begin{array}{c} E\leq \alpha_{1}\leq 1 \end{array}$$

where $E=\frac{{\left(2+\frac{\omega_{1}}{\omega_{2}}\left(\sqrt{\frac{2}{\mu +1}}+1\right)\right)}^{2}}{{\left(2+\frac{\omega_{1}}{\omega_{2}}\left(\sqrt{\frac{2}{\mu +1}}+1\right)\right)}^{2}+\frac{4\omega_{1}}{\omega_{2}\left(\mu +1\right)}}$. In this case, β=γ=1. From Equation (A76) we get that

(A81) $$\begin{array}{c} G_{Alice}=4{\left(\sqrt{\alpha_{1}\omega_{1}}+2\sqrt{\alpha_{2}\omega_{2}}\right)}^{2}-2 \end{array}$$

The derivative of $G_{Alice}$ with respect to $\alpha_{1}$ is given by

(A82) $$\begin{array}{c} \frac{dG_{Alice}}{d\alpha_{1}}=\frac{4\sqrt{2\omega_{1}\omega_{2}}\left(1-2\alpha_{1}\right)}{\sqrt{\alpha_{1}-\alpha_{1}^{2}}}+4\omega_{1}-8\omega_{2} \end{array}$$

The second derivative of $G_{Alice}$ with respect to $\alpha_{1}$ is given by

(A83) $$\begin{array}{c} \frac{d^{2}G_{Alice}}{d\alpha_{1}^{2}}=-\frac{2\sqrt{2\omega_{1}\omega_{2}}}{{\left(\alpha_{1}-\alpha_{1}^{2}\right)}^{\frac{3}{2}}} \end{array}$$

From Equation (A83), we have $\frac{d^{2}G_{Alice}}{d\alpha_{1}^{2}}<0$, and $\frac{dG_{Alice}}{d\alpha_{1}}=0$ has at most one solution when $0\leq \alpha_{1}\leq 1$. By setting $\frac{dG_{Alice}}{d\alpha_{1}}=0$, we get

(A84) $$\begin{array}{c} \frac{1-2\alpha_{1}}{\sqrt{\alpha_{1}-\alpha_{1}^{2}}}=\frac{2\omega_{2}-\omega_{1}}{\sqrt{2\omega_{1}\omega_{2}}} \end{array}$$

Denote $F=\frac{2\omega_{2}-\omega_{1}}{\sqrt{2\omega_{1}\omega_{2}}}$. If F≥0, i.e., $\omega_{1}\leq 2\omega_{2}$, the solution of Equation (A84) is given by $\alpha =0.5-\frac{F}{2\sqrt{F^{2}+4}}=\omega_{1}$. $G_{Alice}$ increases with $\alpha_{1}$ when $\alpha_{1}\in \left(0,\omega_{1}\right)$, and decreases with $\alpha_{1}$ when $\alpha_{1}\in \left(\omega_{1},1\right)$. From $\alpha_{1}\geq E>\omega_{1}$, $G_{Alice}$ gets the maximum at $\alpha_{1}=E$. If F<0, i.e., $\omega_{1}>2\omega_{2}$, the solution of Equation (A84) is given by $\alpha =0.5-\frac{F}{2\sqrt{F^{2}+4}}=2\omega_{2}$. $G_{Alice}$ increases when $\alpha_{1}\in \left(0,2\omega_{2}\right)$, and decreases when $\alpha_{1}\in \left(2\omega_{2},1\right)$. From $\alpha_{1}\geq E>\omega_{1}>2\omega_{2}$, $G_{Alice}$ gets the maximum at $\alpha_{1}=E$. It means that $G_{Alice}$ gets the maximum $G_{Alice_{2}}$ when $x_{2}^{*}\geq 2$, which is further given by

(A85) $$\begin{array}{c} G_{Alice_{2}}=4{\left(\sqrt{\omega_{1}E}+\sqrt{2\omega_{2}\left(1-E\right)}\right)}^{2}-2 \end{array}$$

where the corresponding point is denoted by $p_{2}$.

**Case 3.** For $x_{2}^{*}\leq 0<x_{4}^{*}\leq 1$, it follows that $0<\sqrt{\frac{2\alpha_{1}\omega_{1}}{3\alpha_{2}\omega_{2}}}-\left(\sqrt{\frac{2}{3}}+1\right)\frac{\omega_{1}}{\omega_{2}}\leq 1$ and $\sqrt{\frac{2\alpha_{1}\omega_{1}}{\alpha_{2}\omega_{2}\left(\mu +1\right)}}-\left(\sqrt{\frac{2}{\left(\mu +1\right)}}+1\right)\frac{\omega_{1}}{\omega_{2}}\leq 0$. We get that

(A86) $$\begin{array}{c} D<\alpha_{1}\leq I \end{array}$$

where

(A87) $$\begin{array}{cc} I & =\min(H,G)\\ H & =\frac{{\left(1+\left(\sqrt{\frac{2}{3}}+1\right)\frac{\omega_{1}}{\omega_{2}}\right)}^{2}}{{\left(1+\left(\sqrt{\frac{2}{3}}+1\right)\frac{\omega_{1}}{\omega_{2}}\right)}^{2}+\frac{4\omega_{1}}{3\omega_{2}}}\\ G & =\frac{{\left(\sqrt{\frac{2}{\mu +1}}+1\right)}^{2}\frac{\omega_{1}}{\omega_{2}}}{{\left(\sqrt{\frac{2}{\mu +1}}+1\right)}^{2}\frac{\omega_{1}}{\omega_{2}}+\frac{4}{\mu +1}} \end{array}$$

In this case, we get $\beta =x_{4}^{*}$ and γ=0. From Equation (A76), it follows that

(A88) $$\begin{array}{cc} G_{Alice}= & \frac{4{\left(\sqrt{\alpha_{1}\omega_{1}}+x_{4}^{*}\sqrt{\alpha_{2}\omega_{2}}\right)}^{2}}{\omega_{1}+\omega_{2}x_{4}^{*}}-\mu \alpha_{2}-2\alpha_{1}-\alpha_{2}\beta \\ = & \left(3\sqrt{6}+8\right)\left(\sqrt{\frac{\omega_{1}\alpha_{1}\alpha_{2}}{\omega_{2}}}-\frac{\omega_{1}\alpha_{2}}{\omega_{2}}\right)-2\alpha_{1}\\ \; & -\mu \alpha_{2}+\frac{\omega_{1}\alpha_{2}}{\omega_{2}} \end{array}$$

The derivative of $G_{Alice}$ with respect to $\alpha_{1}$ is given by

(A89) $$\begin{array}{cc} \frac{dG_{Alice}}{d\alpha_{1}}= & \left(3\sqrt{6}+8\right)\left(\frac{\sqrt{\omega_{1}}\left(1-2\alpha_{1}\right)}{\sqrt{8\omega_{2}\left(\alpha_{1}-\alpha_{2}^{2}\right)}}+\frac{\omega_{1}}{2\omega_{2}}\right)\\ \; & -\frac{\omega_{1}}{2\omega_{2}}-2+\frac{\mu }{2} \end{array}$$

The second derivative of $G_{Alice}$ with respect to $\alpha_{1}$ is given by

(A90) $$\begin{array}{c} \frac{d^{2}G_{Alice}}{d\alpha_{1}^{2}}=-\frac{3\sqrt{3\omega_{1}}+4\sqrt{2\omega_{1}}}{4\sqrt{\omega_{2}}{\left(\alpha_{1}-\alpha_{1}^{2}\right)}^{\frac{3}{2}}} \end{array}$$

From Equation (A90), we know that $\frac{d^{2}G_{Alice}}{d\alpha_{1}^{2}}<0$, which implies that $\frac{dG_{Alice}}{d\alpha_{1}}=0$ has at most one solution when $0\leq \alpha_{1}\leq 1$. From $\frac{dG_{Alice}}{d\alpha_{1}}=0$, we get

(A91) $$\begin{array}{c} \frac{1-2\alpha_{1}}{\sqrt{\alpha_{1}-\alpha_{1}^{2}}}=\frac{\sqrt{2}\omega_{2}\left(4-\mu \right)-\omega_{1}\left(6\sqrt{3}+7\sqrt{2}\right)}{\left(3\sqrt{6}+8\right)\sqrt{\omega_{1}\omega_{2}}} \end{array}$$

Denote $J=\frac{\sqrt{2}\omega_{2}\left(4-\mu \right)-\omega_{1}\left(6\sqrt{3}+7\sqrt{2}\right)}{\left(3\sqrt{6}+8\right)\sqrt{\omega_{1}\omega_{2}}}$. The solution of Equation (A91) is given by $\alpha =0.5-\frac{J}{2\sqrt{J^{2}+4}}$. $G_{Alice}$ increases with $\alpha_{1}\in \left(0,\alpha \right)$, and decreases with $\alpha_{1}\in \left(\alpha ,1\right)$. Thus, $G_{Alice}$ gets the maximum at $\alpha_{1}=D$ if α≤D. If α≥I, $G_{Alice}$ gets the maximum at $\alpha_{1}=I$. If D<α<I, $G_{Alice}$ gets the maximum at $\alpha_{1}=\alpha$. The maximum gain of Alice when $x_{2}^{*}<0<x_{4}^{*}<1$ is denoted by $G_{Alice_{3}}$, where the corresponding point is denoted by $p_{3}$.

**Case 4.** For $0<x_{2}^{*}\leq x_{4}^{*}\leq 1$, i.e., $\sqrt{\frac{2\alpha_{1}\omega_{1}}{3\alpha_{2}\omega_{2}}}-\left(\sqrt{\frac{2}{3}}+1\right)\frac{\omega_{1}}{\omega_{2}}\leq 1$ and $\sqrt{\frac{2\alpha_{1}\omega_{1}}{\alpha_{2}\omega_{2}\left(\mu +1\right)}}-\left(\sqrt{\frac{2}{\mu +1}}+1\right)\frac{\omega_{1}}{\omega_{2}}>0$, we get

(A92) $$\begin{array}{c} G<\alpha_{1}\leq H \end{array}$$

If $G_{Bob_{1}}\geq G_{Bob_{2}}$, $\beta =x_{4}^{*}$ and γ=0, the maximum of Alice’s gain $G_{Alice}$ achieves when $0<x_{2}^{*}<x_{4}^{*}<1$. The maximum is denoted by $G_{Alice_{4}}$, where the corresponding point is denoted by $p_{4}$.

**Case 5.** For $G<\alpha_{1}\leq H$ and $G_{Bob_{1}}<G_{Bob_{2}}$, β=0 and $\gamma =x_{2}^{*}$, the maximum of $G_{Alice}$ achieves when $0<x_{2}^{*}<x_{4}^{*}<1$. The maximum is denoted by $G_{Alice_{5}}$, where the corresponding point is denoted by $p_{5}$.

**Case 6.** For $x_{2}^{*}\leq 0$ and $x_{4}^{*}>1$, i.e., $\sqrt{\frac{2\alpha_{1}\omega_{1}}{3\alpha_{2}\omega_{2}}}-\left(\sqrt{\frac{2}{3}}+1\right)\frac{\omega_{1}}{\omega_{2}}>1$ and $\sqrt{\frac{2\alpha_{1}\omega_{1}}{\alpha_{2}\omega_{2}\left(\mu +1\right)}}-\left(\sqrt{\frac{2}{\mu +1}}+1\right)\frac{\omega_{1}}{\omega_{2}}\leq 0$, we get

(A93) $$\begin{array}{c} H<\alpha_{1}\leq G \end{array}$$

In this case, β=1 and γ=0. From Equation (A76), we get

(A94) $$\begin{array}{c} G_{Alice}=\frac{4{\left(\sqrt{\alpha_{1}\omega_{1}}+\sqrt{\alpha_{2}\omega_{2}}\right)}^{2}}{\omega_{1}+\omega_{2}}-\left(\mu +1\right)\alpha_{2}-2\alpha_{1} \end{array}$$

The derivative of $G_{Alice}$ with respect to $\alpha_{1}$ is given by

(A95) $$\begin{array}{c} \frac{dG_{Alice}}{d\alpha_{1}}=\frac{4(\omega_{1}-\frac{\omega_{2}}{2}+\frac{\sqrt{\omega_{1}\omega_{2}}\left(1-2\alpha_{1}\right)}{\sqrt{2(\alpha_{1}-\alpha_{1}^{2})}})}{\omega_{1}+\omega_{2}}+\frac{\mu -3}{2} \end{array}$$

The second derivative of $G_{Alice}$ with respect to $\alpha_{1}$ is given by

(A96) $$\begin{array}{c} \frac{d^{2}G_{Alice}}{d\alpha_{1}^{2}}\leq 0 \end{array}$$

By setting $\frac{dG_{Alice}}{d\alpha_{1}}=0$, we get

(A97) $$\begin{array}{c} \frac{1-2\alpha_{1}}{\sqrt{\alpha_{1}-\alpha_{1}^{2}}}=\frac{\left(7-\mu \right)\omega_{2}-\left(mu+5\right)\omega_{1}}{4\sqrt{2\omega_{1}\omega_{2}}} \end{array}$$

Denote $N=\frac{\left(7-\mu \right)\omega_{2}-\left(mu+5\right)\omega_{1}}{4\sqrt{2\omega_{1}\omega_{2}}}$. The solution of Equation (A97) is given by $\alpha =0.5-\frac{N}{2\sqrt{N^{2}+4}}$. $G_{Alice}$ increases with $\alpha_{1}\in \left(0,\alpha \right)$, and decreases with $\alpha_{1}\in \left(\alpha ,1\right)$. Thus, if α≤H, $G_{Alice}$ gets the maximum at $\alpha_{1}=H$. If α≥G, $G_{Alice}$ gets the maximum at $\alpha_{1}=G$. If H<α<G, $G_{Alice}$ gets the maximum at $\alpha_{1}=\alpha$. The maximum of $G_{Alice}$ achieves when $x_{2}^{*}<0$ and $x_{4}^{*}>1$, and is then denoted by $G_{Alice_{6}}$, where the corresponding point is denoted by $p_{6}$.

**Case 7.** For $0<x_{2}^{*}<1<x_{4}^{*}$, i.e., $\sqrt{\frac{2\alpha_{1}\omega_{1}}{3\alpha_{2}\omega_{2}}}-\left(\sqrt{\frac{2}{3}}+1\right)\frac{\omega_{1}}{\omega_{2}}>1$ and $0<\sqrt{\frac{2\alpha_{1}\omega_{1}}{\alpha_{2}\omega_{2}\left(\mu +1\right)}}-\left(\sqrt{\frac{2}{\mu +1}}+1\right)\frac{\omega_{1}}{\omega_{2}}<1$, we get

(A98) $$\begin{array}{c} R<\alpha_{1}<Q \end{array}$$

where $Q=\frac{{\left(1+\frac{\omega_{1}}{\omega_{2}}\left(\sqrt{\frac{2}{\mu +1}}+1\right)\right)}^{2}}{{\left(1+\frac{\omega_{1}}{\omega_{2}}\left(\sqrt{\frac{2}{\mu +1}}+1\right)\right)}^{2}+\frac{4\omega_{1}}{\omega_{2}\left(\mu +1\right)}}$ and R=max(H,G). If $G_{Bob_{3}}\geq G_{Bob_{4}}$, β=1 and γ=0, the maximum of $G_{Alice}$ achieves when $0<x_{2}^{*}<1<x_{4}^{*}$, and is then denoted by $G_{Alice_{7}}$, where the corresponding point is denoted by $p_{7}$.

**Case 8.** For $R<\alpha_{1}<Q$ and $G_{Bob_{3}}<G_{Bob_{4}}$, β=0 and $\gamma =x_{2}^{*}$, the maximum of $G_{Alice}$ achieves when $0<x_{2}^{*}<1<x_{4}^{*}$, and is then denoted by $G_{Alice_{8}}$, where the corresponding point is denoted by $p_{8}$.

**Case 9.** For $1\leq x_{2}^{*}<2$, i.e., $1\leq \sqrt{\frac{2\alpha_{1}\omega_{1}}{\alpha_{2}\omega_{2}\left(\mu +1\right)}}-\left(\sqrt{\frac{2}{\mu +1}}+1\right)\frac{\omega_{1}}{\omega_{2}}<2$, we get

(A99) $$\begin{array}{c} Q\leq \alpha_{1}<E \end{array}$$

In this case, $\beta =x_{2}^{*}-1$ and γ=1. From Equation (A76), we get

(A100) $$\begin{array}{cc} G_{Alice}= & 4\frac{{\left(\sqrt{\alpha_{1}\omega_{1}}+x_{2}^{*}\sqrt{\alpha_{2}\omega_{2}}\right)}^{2}}{\omega_{1}+\omega_{2}x_{2}^{*}}-2\alpha_{2}-2\alpha_{1}-\alpha_{2}x_{2}^{*}\\ = & \left(2\sqrt{2(\mu +1)}+3\sqrt{\frac{2}{\mu +1}}+8\right)\\ \; & \times \left(\sqrt{\frac{\omega_{1}\alpha_{1}\alpha_{2}}{\omega_{2}}}-\frac{\omega_{1}\alpha_{2}}{\omega_{2}}\right)-2\alpha_{1}-2\alpha_{2}+\frac{\omega_{1}\alpha_{2}}{\omega_{2}} \end{array}$$

The derivative of $G_{Alice}$ with respect to $\alpha_{1}$ is given by

(A101) $$\begin{array}{cc} \frac{dG_{Alice}}{d\alpha_{1}}= & \left(2\sqrt{2(\mu +1)}+3\sqrt{\frac{2}{\mu +1}}+8\right)\\ \; & \times \left(\frac{\sqrt{\omega_{1}}\left(1-2\alpha_{1}\right)}{\sqrt{8\omega_{2}\left(\alpha_{1}-\alpha_{2}^{2}\right)}}+\frac{\omega_{1}}{2\omega_{2}}\right)-\frac{\omega_{1}}{2\omega_{2}}-1 \end{array}$$

The second derivative of $G_{Alice}$ with respect to $\alpha_{1}$ is given by

(A102) $$\begin{array}{c} \frac{d^{2}G_{Alice}}{d\alpha_{1}^{2}}=-\frac{2\sqrt{2\omega_{1}\left(\mu +1\right)}+3\sqrt{\frac{2\omega_{1}}{\mu +1}}+8\sqrt{\omega_{1}}}{\sqrt{32\omega_{2}{\left(\alpha_{1}-\alpha_{1}^{2}\right)}^{3}}} \end{array}$$

From Equation (A102), we gave $\frac{d^{2}G_{Alice}}{d\alpha_{1}^{2}}<0$ which implies that $\frac{dG_{Alice}}{d\alpha_{1}}=0$ has at most one solution when $0\leq \alpha_{1}\leq 1$. From $\frac{dG_{Alice}}{d\alpha_{1}}=0$, we get

(A103) $$\begin{array}{c} \frac{1-2\alpha_{1}}{\sqrt{\alpha_{1}-\alpha_{1}^{2}}}=\frac{\left(4\omega_{2}-14\omega_{1}\right)\sqrt{\mu +1}-\sqrt{2}\omega_{1}\left(4\mu +10\right)}{\left(4\mu +10+8\sqrt{2}\sqrt{\mu +1}\right)\sqrt{\omega_{1}\omega_{2}}} \end{array}$$

Denote $T=\frac{\left(4\omega_{2}-14\omega_{1}\right)\sqrt{\mu +1}-\sqrt{2}\omega_{1}\left(4\mu +10\right)}{\left(4\mu +10+8\sqrt{2}\sqrt{\mu +1}\right)\sqrt{\omega_{1}\omega_{2}}}$. The solution of Equation (A103) is given by $\alpha =0.5-\frac{T}{2\sqrt{T^{2}+4}}$. $G_{Alice}$ increases with $\alpha_{1}\in \left(0,\alpha \right)$, and decreases with $\alpha_{1}\in \left(\alpha ,1\right)$. Thus, if α≤Q, $G_{Alice}$ gets the maximum at $\alpha_{1}=Q$. If α≥E, $G_{Alice}$ gets the maximum at $\alpha_{1}=E$. If Q<α<E, $G_{Alice}$ gets the maximum at $\alpha_{1}=\alpha$. The maximum value of $G_{Alice}$ achievers when $1<x_{2}^{*}<2$, and is then denoted by $G_{Alice_{9}}$, where the corresponding point is denoted by $p_{9}$.
